# Lessons From Special Forces Operators for Elite Team Sports Training: How to Make the Whole Greater Than the Sum of the Parts

**DOI:** 10.3389/fspor.2022.780767

**Published:** 2022-03-21

**Authors:** Nathalie Pattyn, Jeroen Van Cutsem, Emilie Lacroix, Martine Van Puyvelde, Aisha Cortoos, Bart Roelands, Veerle Tibax, Emilie Dessy, Magali Huret, Gerard Rietjens, Maarten Sannen, Robert Vliegen, Jean Ceccaldi, Jérémy Peffer, Ellen Neyens, Nathalie Duvigneaud, Damien Van Tiggelen

**Affiliations:** ^1^VIPER Research Unit, LIFE Department, Royal Military Academy, Brussels, Belgium; ^2^Human Physiology and Sports Physiotherapy Research Group, Vrije Universiteit Brussel, Brussels, Belgium; ^3^Brain Body and Cognition Research Group, Department of Psychology and Educational Sciences, Vrije Universiteit Brussel, Brussels, Belgium; ^4^Brainwise Ltd, Overijse, Belgium; ^5^Directorate General Human Resources, Department of Defence, Brussels, Belgium; ^6^Centre for Mental Health, Military Hospital Queen Astrid, Brussels, Belgium; ^7^Korps Commandotroepen, Dutch Defence, Roosendaal, Netherlands; ^8^Special Forces Group, Belgian Defence, Brussels, Belgium; ^9^Medical Regional Centre in Beauvechain Air Base, Belgian Defense, Brussels, Belgium; ^10^Centre for Physical Medicine & Rehabilitation, Military Hospital Queen Astrid, Brussels, Belgium; ^11^Department Rehabilitation Sciences, Faculty of Medicine & Health Sciences, Ghent University, Brussels, Belgium

**Keywords:** team performance, special forces, multidisciplinary, team sports, sports physiotherapy, performance psychology

## Abstract

This methodology paper describes the design of a holistic and multidisciplinary human performance program within the Belgian Special Forces Group, the Tier 1 Special Operations unit of the Belgian Defense. Performance management approaches in the military draw heavily on sports science. The key component of the program design described here is its integrative nature, which team sports training might benefit from. The basic rationale behind the program was to bridge several gaps: the gap between physical and mental training; the gap between the curative or preventive medical approach and the performance enhancement approach; and the gap between individual and team training. To achieve this goal, the methodology of Intervention Mapping was applied, and a multidisciplinary team of training and care professionals was constituted with operational stakeholders. This was the first step in the program design. The second step took a year, and consisted of formal and informal consultations, participant observations and task analyses. These two first stages and their conclusions are described in the Method section. The Results section covers the next two stages (three and four) of the process, which aimed at defining the content of the program; and to test a pilot project implementation. The third stage encompassed the choice of the most relevant assessment and intervention tools for the target population, within each area of expertise. This is described extensively, to allow for replication. The fourth and last stage was to “test drive” the real-life integration and implementation of the whole program at the scale of a single team (8 individuals). For obvious confidentiality reasons, the content data will not be reported extensively here. Implications for wider-scale implementation and tie-back to sports team training are presented.

## Introduction

### Context: How Sports Science and Special Operations Support Relate

Special Operations differ from conventional military operations in “the degree of physical and political risk, operational techniques, modes of employment, independence from friendly support, and dependence upon detailed operational intelligence, ” as described by Day and Horn (2010). Accordingly, Special Operations Forces (SOF) personnel require skills and abilities above and beyond those from members of the general body of the military, sometimes to the point of contradiction: whereas a conventional member is expected to fit in an overall framework and hierarchical structure governed by a well-defined set of rules, a SOF operator is expected to be able to switch between the latter, and the ability to creatively think out of the box in an extremely self-sufficient manner when all well-defined rules have failed. Indeed, SOF personnel are expected to be able to function in small teams, self-reliant, and independent, hereby making the team the smallest unit of performance of such structures.

The Special Forces Group (SF Gp) is the Tier 1 SOF (Special Operations Forces) unit of the Belgian Defense. Its members are selected rigorously on mental and physical capacity and the qualification course to join lasts for 6 months. It is the military training where attrition is the highest (around 80%), yet only the first quarter of the grueling complete training of an operator. This lengthy selection is based on the search for extreme physical fitness and endurance, intrinsic motivation, and mental toughness (Pattyn and Vliegen, 2019). The essence of the unit is simple: technical skills, physical endurance and mental strength make the difference between life and death; therefore, training is never complete. Operators are thus, by definition, a very limited number of elite military personnel with a very high operational readiness level. In 2017, the unit set out to design a customized human performance program for its members. The current paper is a report of the design and implementation of this program.

Despite the fact that several scientific publications (e.g., Farina et al., 2019) underscore the multidimensional (as in, linking physiology and psychology) nature of a successful selection of suitable candidates, the training and monitoring of the SF “tactical athlete” is still largely looked upon as a dichotomy: strength and conditioning for the healthy, medical rehabilitation for the injured. As underscored by Lunasco et al. (2019) regarding a new Human Performance Optimization (HPO) concept for the US SOF community: “HPO is defined as the process of applying knowledge, skills, and emerging technologies to improve and preserve the capabilities of SOF personnel to execute mission essential tasks[…]. If we adopt this framework, however, we must move beyond the current illness-based model of care and adopt or create a more suitable structure and scope of practice.”

**Figure 1:**
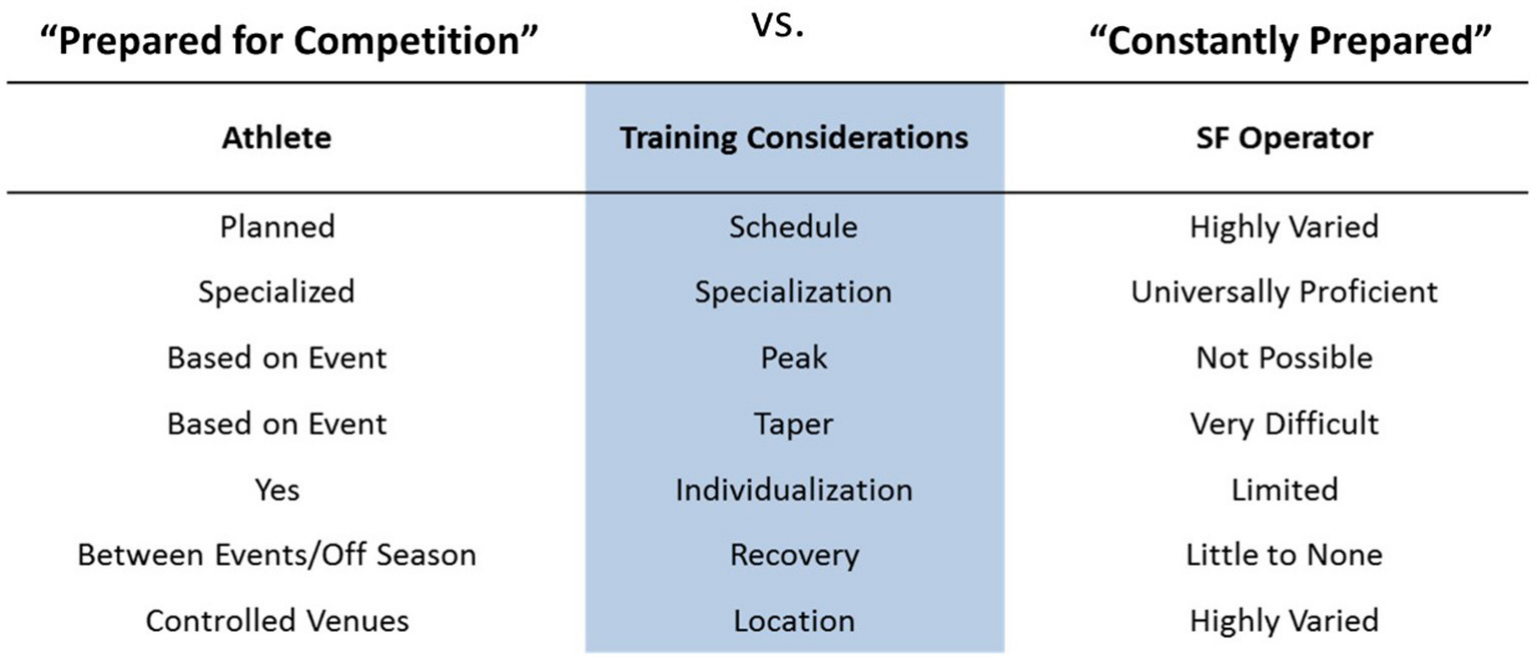
Summary of the differences regarding training between elite athletes and SOF operators. Adapted from Solberg (2017).

Performance enhancement coaching is another area where SOF support needs to draw on sports science. Whereas, the performance enhancing aspects of coaching have been known in the sports world for decades, the first formalized program in SOF communities has been publicized in 2017 (Barry and De Vries, 2019), which is remarkably late. Mattie et al. (2020) were the first to report the design and implementation of a specific mental skills training program for a SOF environment. Training and performance management in SOF support are usually physiology-centric, and based on training models for elite athletes. However, differences between athletes and operators have already been identified and summarized (see Figure 1).

The two main differences regarding training between an elite athlete and a SOF operator are, firstly, the fact that an elite athlete can focus on a very narrow spectrum of performance related to his/her discipline, whereas a SOF operator needs to excel in a multitude of domains; and secondly, the fact that the tapered training of an athlete, who works up his/her performance toward important milestones does not apply to an operator, who needs to be ready all year round. Operators are athletes who cannot follow a periodised or fixed training schedule for a single type of physical activity, such as other professional athletes. In this regard, the athletes that are closest to operators regarding training demands are those practicing team sports. The all-round type and all-year-round readiness athletic profile of the operator requires specific skills and readiness. Regarding the physical performance, primary, and secondary musculoskeletal injury prevention is paramount and therefore a tailored, and more importantly, an individualized screening and corrective training/rehabilitation program is needed. Again, similarly to team sports, where a team usually comprises members with a previous medical history of wear and tear musculoskeletal pathology, the approach to training for operators needs to take into account this injury management as well, in function of individual vulnerabilities. In order to achieve this individual adaptation, a musculoskeletal screening needs to comprise the joint mobility, flexibility, neuromuscular control, muscular endurance, and strength. The purpose is to get data to adapt individual training and perform an individualized follow-up during the whole operator's career. Regarding the mental performance, the rationale is similar: the specific demands of the year-round readiness in cognitively and emotionally demanding situations require specific skills and a specific mindset, developed and followed-up at an individual level, taking into account each person's strengths and weaknesses. This paper describes the detailed screening and assessment methods used in the Belgian Special Forces Group to develop a tailored human performance management program.

### Rationale: Should Performance Management Start With Overcoming Duality?

Traditionally, both in military elite units and in elite sports science, enhancing performance has focused on the physical component of performance by relying on exercise physiology: how to boost training in order to allow for the individuals to overcome the limitations of human physiology, both in terms of strength and endurance. In the past decades, both environments have acknowledged a growing role for the mental component of performance.

The mind-body opposition in our Western tradition of thought can be traced back to the French philosopher René Descartes in the XVIIth century. It was in 1641 (Descartes, in Khodoss, 2004) that cartesian dualism was formalized by its author, creating a rift between mind and body, with which we are still struggling nowadays (Pattyn, 2009). Despite “mental fatigue” being the latest revolution in exercise physiology (for a review of how this demonstrates a lag compared to other research fields, see Pattyn et al., 2018), training and coaching still rely heavily on physiology, as demonstrated by sports science curricula, the composition of multidisciplinary teams surrounding professional athletes, or even the amount of training devoted to psychological skills (e.g., Otte et al., 2020).

The mind-body opposition is not the only dualism plaguing performance management. There are two others which are even lesser known and acknowledged. The second one is the dichotomy between “injured/ill” and “healthy.” Current models of performance management often place performance optimization at the extreme end of good health (e.g., Lunasco et al., 2019), hence sayings like “being in the blue” or “staying left of boom, ” referring to the spatial orientation or the color coding of this continuum. However, this is based on the assumption that performance is an enhanced state of health. Looking at evidence both from elite soldiers and top athletes, it shows clearly that high performers can have a pathology category of their own, both on the physiological and psychological dimensions, without even touching upon the potential intrinsic pathology of overachievement (e.g., Nixdorf et al., 2015).

The third opposition is not readily perceived as such, but it is a dichotomy where it should be a continuum: the opposition between the individual and the team, when it comes to selection, diagnostics, monitoring, and interventions. On the one hand, there is a growing interest in the medical field for customization and individual differences approach, with personalized medicine being the epitome of this evolution. On the other hand, there is a whole body of research on team performance, with a systemic approach of team roles and team cognition; and this area being a research and expertise topics in its own right (e.g., in aviation or the military). However, there is still a gap to bridge between those two extremes. As some recent publications from the sports science field emphasize: we might need to move away from training and toward synergizing our elite teams (e.g., Soltanzadeh and Mooney, 2016; Pol et al., 2020). This holds true for both SOF support and sports science (and any high-performance environment where the smallest unit of performance is not an individual but a team).

### Team Training in Sports Science: From the Individual Athlete to the Team

Sport training is the process of systematically performing exercises to improve physical and cognitive abilities and to acquire specific sport skills (Impellizzeri et al., 2019). When delivered appropriately, training produces a functional adaptive response that induces shifts in various training outcomes such as physical, technical and/or tactical performance, injury resistance, or health (Impellizzeri et al., 2019). Important differences in training philosophy and approach arise when individual and team sports are compared. An athlete practicing an individual sport often peaks toward one or more short competition periods, whereas typically team sport players are required to perform throughout an entire (and long) season, which is more similar to the level of readiness required from operators. The divergent goal set for the individual athlete or the team infers training content and schedules to be adapted toward achieving that goal. Hence, there is an important role for planning and periodization, recovery (internal and external), load monitoring, and medical follow-up. In team training, variation and periodization are widely acknowledged as crucial to optimizing the training responses (Gamble, 2006). Training variation is an absolute must to alleviate the monotony that can otherwise affect compliance throughout a long season of training and competition (Gamble, 2006). In general, periodization in team sports has been described as built up of several phases, starting with a mandatory preparatory phase, a competitive phase, and a transition phase in which emphasis is on full physical and mental recovery after a competitive season (Lyakh et al., 2016). Team sports coaches face an additional issue with the integration of different goal sets. The variety of training goals throughout a season (such as important games for the club; international games) and between the players (depending on position on the field and current state of fitness or injuries), as well as the extended duration of competition, pose unique challenges to periodized planning (Gamble, 2006).

In recent years the concept of training has been, and still is, revisited. The general—reductionist—idea of training has always been that the individual athletes within the team should be maximally trained, leading to an optimal performance. If the same closed recipe was applied to all of the players who formed part of the team, global team performance would also be optimal (Sainz, 2020). It could however be postulated that teams should be viewed as complex adaptive systems, whose behavior evolves in response to physical and informational constraints. From this perspective, athletes and teams are conceptualized as dynamic complex systems interacting non-linearly, i.e., co-adaptively, with the environment (Pol et al., 2020). Team performance cannot be determined by summing up performance levels of the individuals composing the team (Soltanzadeh and Mooney, 2016). This innovative approach in team sports training has so far only been described at a conceptual level in the scientific literature. The current paper presents its first practical application within a SOF team.

To conclude, we set out to tailor a human performance program aimed at overcoming the dichotomy between mental and physical performance; between care for existing injuries and performance optimization; and between individual training and team functioning. In the next “Methods” section, we will detail how we conceived this integrated program. As this conception entailed preliminary observation and investigations, the results of these phases are discussed in the “Method” section. In the “Results” section, we will describe the domain specific tools we applied and how we implemented them. The integration of all the concepts to reach the goal we aimed for will provide a multidisciplinary human performance program that allows for a holistic approach of the individual, not limited to a curative framework and considering the team as the smallest unit of performance of the system.

## Method

### Methodology for Program Design: Intervention Mapping

The design and implementation of a Mental Skills Training program within the Canadian SOF was described by Mattie et al. (2020) using Intervention Mapping (IM, Bartholomew Eldridge et al., 2016). IM is a method comprising six steps: (1) needs assessment, which can range from literature review to focus group consultation or participating observations; (2) identification of program outcomes and objectives, i.e., the “what do we want” stage; (3) program design, which in our case covered the selection of theory-driven and evidence-based methods and practical strategies from each professional background; (4) program production including pilot testing, which allowed to finetune and adapt the chosen methods along the way; (5) planning for adoption, implementation and sustainability, and (6) program evaluation. As emphasized by Mattie et al. (2020), IM is a valuable framework for the development of customized training and support programs for military personnel. One of the major added values of this approach is the transparency of the process, and the integration of relevant theory and evidence into program development. Furthermore, since a co-creation with the end-user was of paramount importance to us, both for ethical and practical reasons, this methodological framework seemed ideal in facilitating ongoing consultation with the end-user, hence enhancing the chances of effective implementation and user acceptance.

The current paper will cover the four first steps, which are summarized in Table 1. This method section will cover the two first steps and describe the needs assessment and the program objectives, which will thus include the results from these steps. The “Results” section will cover step” and step 4, and describe the practical program design, including all the necessary information for replication (i.e., the kitchen recipe); step 4 being the blueprint of implementation in a pilot project. As we will detail in the discussion, the current paper only covers step 1 to step 4. This approach and its description allow for an optimal transparency in the report of our design process: our aim is to present what we designed (the kitchen recipe) and why and how we designed it (hence providing the rationale behind each choice).

**Table 1:** Overview of the four first steps of the Intervention Mapping methodology applied to the current project design.

**Methods**	
STEP 1 Needs assessment	• Establish a multidisciplinary expert and stakeholders team to design the program. • Determine the current needs based on real-life participant observation and analysis.
STEP 2 Determine program objectives	• Setting-up the program within a holistic approach regarding health and performance. • Define an individualized tailor-made approach to customize the whole support. • Address physical activity, nutrition and sleep needs to facilitate healthy lifestyle choice and performance improvement. • Support injury prevention and healthy coping mechanisms.
**Results**		
STEP 3 Program design according to each area of expertise	• Physiotherapy ° Identify body regions discomfort and potential musculoskeletal injuries through a first screening questionnaire. ° Provide an overall whole body functional movements assessment. ° Offer a detailed assessment for specific injuries involving lower back, cervical or lower/upper limb dysfunctions. • Physical training ° Define a detailed individualized physical performance assessment. · Provide a specific, validated and practical test battery · Create an evaluation tool to be used by the PTI, the operator and the physiotherapists. ° Provide individualized physical training programs. ° Adapt specific nutrition and hydration knowledge to the particular constraints of the population. • Performance psychology ° Determine the specific psychometry assessment need. ° Specify the most adequate validated trait and state assessment tools. ° Provide a customized individual feedback. ° Dispense a team workshop to provide feedback and determine possible interventions
STEP 4 Implementation in a pilot project	• Conceive a modular training program about the impact of human factors on the individual and team functioning. • Integrate an evolution from individual functioning to team functioning; and from participant operator receiving expert advice to autonomous actor of their own performance management. • Distribute the program throughout the year, according to the modular built-up principles discussed earlier: ° Four weeks at the unit (January – April – June- December) ° Two deployment periods (3 weeks/3 months) with embedded experts.

*Step 4 is further detailed in Table IV*.

### Step 1: Needs Assessment

#### Team Composition to Design the Program

As described in the introduction, one of our core assumptions was the need for true multidisciplinarity within the program, as from the start. Furthermore, we applied the principles of system theory in the design of the program: the support to be offered could not be defined by external experts only, but had to be a non-hierarchical co-creation between the expert and the actual “client” (McTaggart, 1991; McIntyre, 2007; Gergen and Gergen, 2008), being in this case the unit in itself, and more specifically, the active operators (Soltanzadeh and Mooney, 2016).

The choice of the multidisciplinary professional experts to be included in the design and implementation team was based on three criteria: (i) professional expertise in the core specialty; (ii) relevant operational military experience allowing for an efficient leverage of said professional expertise; and (iii) choice of the unit. The third criterion is subjective, yet of paramount importance to build the trust relationship necessary in this process. These experts comprised one medical doctor (MD), one performance psychologist, one clinical psychologist, three physiotherapists, and one exercise physiologist. The operational members of the unit who participated in the program design were the RSM (Regiment Sergeant Major), who is the senior NCO of the unit, and who at that time was the operator with the longest operational career in the unit; the senior medic, who was also an active operator; the unit's physical training instructor (PTI); and the team leader of the team who volunteered for the pilot project implementation.

#### Observation and Analysis

An international benchmarking consultation was initiated by the project team, in order to identify successful strategies in partner countries, and request support in the program design where available. In 2017, CANSOFCOM, the Canadian Special Operations Forces Command hosted a Human Performance Symposium aiming to provide this international benchmarking and collaborative networking. Following the attendance and networking, support was requested and obtained from the Netherlands, regarding the design of the physical assessment, training, and nutrition part; and from Canada regarding program design and implementation on the one hand; and mental performance on the other hand. In the meantime, the Canadian team published their program design approach in 2020 (Mattie et al., 2020).

Rather than limiting the activities of the design team to consultation meetings, which we feared would have created the risk of disconnecting the design process from the shop floor experience of the operators, we chose to anchor the process in the reality of the unit, through participant observation (Jorgensen, 1989; Spradley, 2016). Over the course of 1 year, several of the professional experts (only those with an active duty status) were included in operational deployments (exercises, courses, and actual missions) of the unit. These included portions of the qualification course, the counter-terrorism course, the personal and vehicle security course, two international exercises, and one mission in a conflict zone. Each professional fulfilled a support function during these observations, to feed the program development with real-life needs assessments.

The first issue which was consistently named by all consulted members of the unit regarding their performance management was the prevalence of musculoskeletal injuries. The physiotherapists thus conducted an in-depth screening with 60% of all active operators to quantify the problem and thus inform the program design. From all the screened operators, 88% showed either chronic or recurrent musculoskeletal issues, despite their active duty professional fitness qualification. This “active duty fitness” is a highly demanding occupational medical screening, aimed at medical risk mitigation for frequently deployed personnel, especially in combat functions. The prevalence information regarding musculoskeletal injuries was only obtained because of the confidentiality guarantee toward the operators. The further description of assessment and diagnosis is detailed in the “Results” section.

One of the support functions which was the least used and known by the active operators at the beginning of this process was clinical psychology. In order to define in which way this function could answer the needs, an exploratory survey was conducted through individual interviews with 52% of all active operators within the unit. This allowed to have a better grasp on the risk factors for their mental health and well-being from their own perspective. The results of this survey have been published elsewhere (Huret, 2018), however, they were recently confirmed by Frueh et al. (2020), in their results describing the medical and behavioral healthcare needs of a special forces population in the US military.

### Step 2: Program Objectives

The second step of the IM approach involves determining the desired outcomes that should occur as a result of program implementation. In this definition stage of the program objectives, a first field of tension was identified, in the question whether the program was actually advocating for individuals' interests (i.e., the active operators) or institutional interests (the unit or on the larger scale the Department of Defense). This ethical issue will be further addressed in the discussion. For the current program development, the choice was made to always prioritize the interests of the individual operators. A practical example was the confidentiality of the results of assessments made in the framework of the program. From the start, the design team determined that those results could not be part of the official medical service record of the operators, in order to preserve the trust relationship to be built with operators.

Program objectives are defined here as design requirements, not as quantifiable performance indicators for the outcome in terms of operators' performance. The difference lies in what in medico-legal terms is defined as an obligation of means vs. an obligation of results. The program design objectives are thus defined regarding what the project team identified as requirement for the program, based on the needs assessment.

#### A Holistic Program

According to the operators, being one is not a profession, it is a way of life. Hence the necessity, as acknowledged by other programs (e.g., Lunasco et al., 2019) to address mental and physical health and well-being, in both the professional and personal aspects of life. This further supports our initial choice of multidisciplinarity; and of the systemic approach regarding mental health. This aspect holds true for elite athletes as well: the commitment to training and performance is of such magnitude that it does not allow for a compartmentalized life.

#### Capitalize on Strengths

From an institutional point of view, a defining feature of the Belgian unit compared to partner nations is its small size. Whereas, this could be viewed as a weakness, in terms of availability of resources and leverage, it is also a strength, as it allows for an individualized approach, customizing the whole support offer to the specific needs of each individual and each team. This is also the case in professional team sports, where the support teams knows each athlete individually, and where the whole work is organized in a tailored fashion to that specific setting. As such, our experience might be more relevant to sports team training than that of larger nations, where the “client population” amounts to hundreds of people.

From an individual point of view, defining features of an operator emerged from our consultations and observations performed during Step 1: an individual with an exceptionally high need for achievement, sense of self-discipline and need for autonomy (Huret, 2018). These features need to be leveraged as cornerstones of the approach; and further justify our original choice of early stakeholder involvement considering the need for autonomy of operators.

#### Facilitate Healthy Lifestyle Choice, Sustaining Performance Improvement

We identified a 3-fold gap in the application of the World Health Organization's “Pillars of Health” (i.e., physical activity, nutrition, and sleep): lack of education, lack of support and guidance, and lack of material availability (as in healthy food, equipment or space for training, reach back to experts providing guidance etc). Physical activity is so paramount in both the function of the operator but also his coping mechanism (Huret, 2018) that we addressed it separately. Regarding nutrition and sleep, as there were no institutional programs within Defense in these areas at the time, we distributed them within the experts team: nutrition would be addressed by the Dutch exercise physiologist consulting for the program (who had the expertise of implementing a nutrition optimization program within the Dutch SOF unit, e.g., Rietjens et al., 2021), the MD and the physical training instructor (PTI); and sleep would be addressed by the MD (who had the expertise of decades of research regarding sleep in extreme environments, e.g., Pattyn et al., 2018) and the performance psychologist. Lack of education was addressed in our pilot project, as well as lack of support and guidance. Lack of material availability could not be fully met, as procurement (of equipment or food for example) may be well above the level of responsibility of the unit. The detailed program to address these issues is summarized in Step 4.

#### Address Vulnerabilities

The multiple stressors and professional demands to which operators are subjected have been extensively described elsewhere (e.g., Huret, 2018; Pattyn and Vliegen, 2019; Mattie et al., 2020). However, two main intervention axes were identified in the needs assessment (which was the first step of our program design): the physical wear and tear, sometimes described as the “shelf-life” of the operator; and the existing coping mechanisms to deal with these exceptional stressors. Since these intervention axes were guiding red threads in the design of our program objectives, we describe them in this section.

##### Physical Wear and Tear

The staggering prevalence of 88% for musculoskeletal issues has already been mentioned. Considering the fact that physical performance is a basic requirement for the job of operator, injury prevention and management has to be a key component of the program. Furthermore, for the personal well-being and overall life satisfaction of operators, this ability to function pain free in their personal life (carrying children, practicing sports, transitioning to another profession without physical limitations) is a major concern.

##### Support Existing Coping Mechanisms

Operators thrive in a context where many “normal” individuals would feel uncomfortable. Therefore, part of the initial survey regarding mental health and attitudes toward psychological support was to map their coping mechanisms, in order to identify which could be further supported. Three main factors of healthy coping mechanisms were identified in the individual interviews: sports practice (for 100% of the interviewed operators), partner relationships (88%), and team dynamics (50%). For the program design, we thus focussed on developing support for these three domains. Furthermore, considering the specificity of the SOF professional environment, the reliance on the team as the basic unit of functioning, and the long durations of deployments for courses, exercises and missions, it was important to make members partly self-sufficient in their performance management. As we already emphasized previously: the need for autonomy is one of the core features of individuals in SOF, hence our leveraging of this feature in our program design approach. Similarly to what is implemented regarding medical care (NATO SOF Medical standards and training directive, 2009), where operators have the most in-depth training for non-medical personnel regarding technical procedures and access to pharmacological treatments, because of their need to function in a self-sufficient manner, we set out to design tools that could enable them, once a period of basic psycho-education and implementation testing was fulfilled, to provide the first line of care for themselves.

The next section, “Results, ” will describe the two next steps in our IM project design: the actual fulfillment of these requirements, with the practical content of the program design. Since the approach for this program was to map individual strengths and weaknesses to build a team training upon, the first step was to select the relevant individual assessment to work with. The next section will describe these assessments in detail, as well as the rationale behind the respective choices.

## Results

### Step 3: Program Design According to Each Area of Expertise

#### Physiotherapy

**Figure 2:**
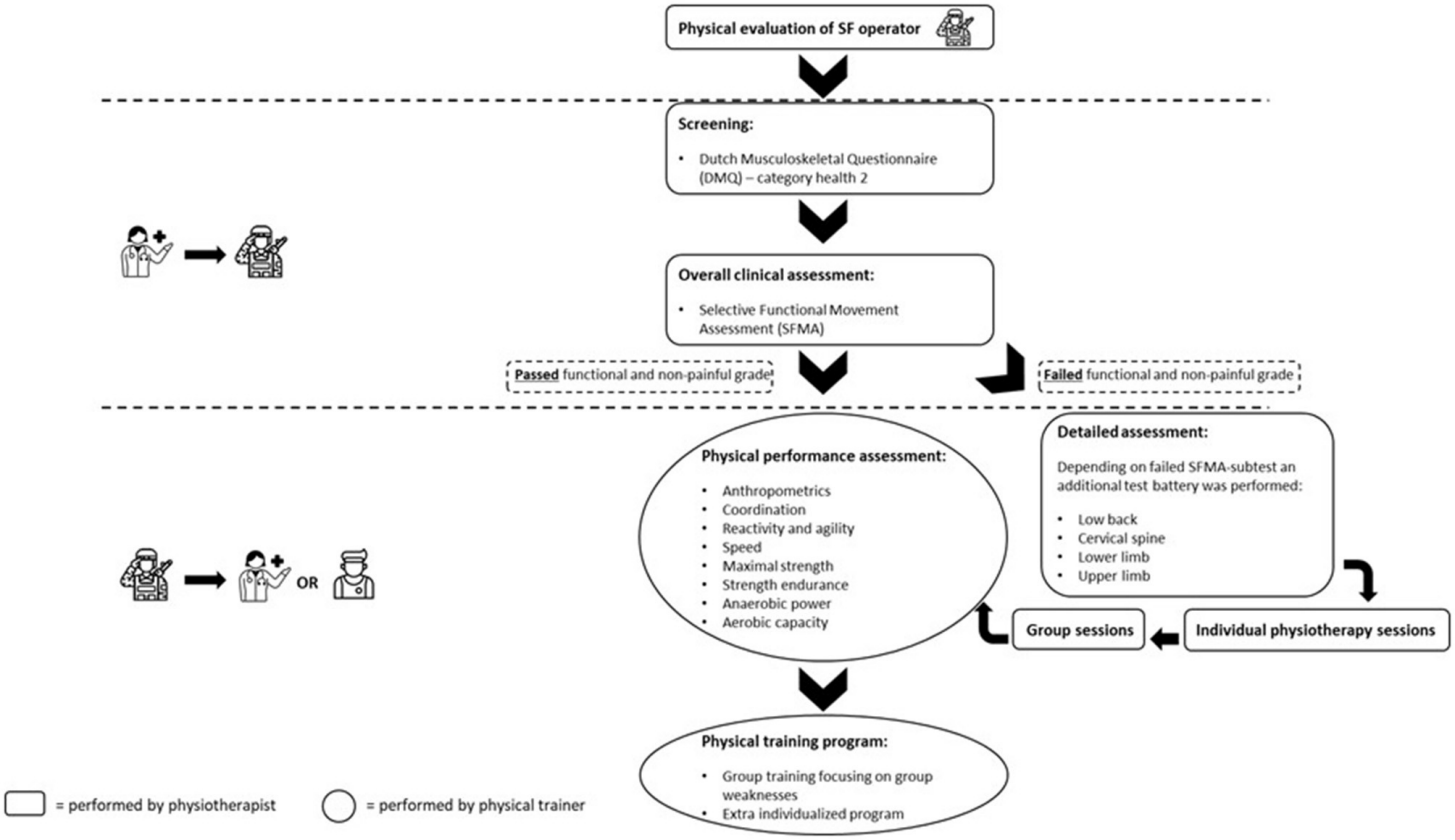
Flow chart of the physiotherapy assessment and transition to the physical training instructor evaluations. “Screening” and “Overall clinical assessment” are performed by the physiotherapist in the unit, “Detailed assessment” in the Center for Physical Medicine and Rehabilitation of the Military Hospital Queen Astrid and the “Physical performance assessment” is done by the unit physical training instructor.

According to the framework discussed before, the physiotherapy approach is centered around individualization. A first challenge resided in the combination of injury prevention and treatment on the one hand; and performance enhancement on the other hand. Considering the fact that 88% of all screened operators in our population reported chronic musculoskeletal issues, this clinical approach is necessary to allow for a continued physical training without enhancing the existing problems. To allow for a rational workload distribution, the assessment of operators is layered and modular, which we will detail below (see also Figure 2).

##### Layer 1: Screening

Operators completed a short version of the Dutch Musculoskeletal Questionnaire (DMQ) category health 2, to identify the body regions with pain or discomfort (Hildebrandt et al., 2001; Southerst et al., 2013). As previous injuries are the utmost important risk factor for future injuries, this history is carefully recorded (Fulton et al., 2014). Post-injury assessments demonstrate for example modifications in strength, proprioception, motor control and even kinematics, which are known risk factors of clinical importance in sports injury prevention (Parr et al., 2015).

##### Layer 2: Overall Clinical Assessment Through Selective Functional Movement Assessment

Impairments associated with musculoskeletal injuries are rarely confined to the injured joint, and residual deficits can persist if these impairments are not addressed properly. These deficits and remote dysfunctions are not easily identifiable by traditional, joint-specific examination techniques. Therefore, we selected the Selective Functional Movement Assessment (SFMA), a movement-based functional assessment model, to be performed on all operators to identify weak links affecting overall functions and estimate injury risk (Glaws et al., 2014; Goshtigian and Swanson, 2016). Data were collected by trained military physiotherapists from the Military Hospital Queen Astrid (Brussels, Belgium) to uncover insights about actual and former pain experiences and functioning. Clinical tools like SFMA that incorporate whole body functional movements may uncover important underlying impairments that allow for the development and implementation of targeted interventions to both maximize recovery after primary injury and prevent secondary injury (Glaws et al., 2014).

With the SFMA the therapist assesses posture, muscle balance, and movement patterns in order to identify relevant musculoskeletal dysfunction in a clinical population. It guides physiotherapists to dysfunctional movements not seen with more conventional examination procedures (Goshtigian and Swanson, 2016; Fauntroy et al., 2019).

Using the SFMA Categorical Scoring tool, each basic Top Tier test is graded as Functional and Non-painful, Functional Painful, Dysfunctional Non-painful or Dysfunctional Painful. If a Top Tier test does not pass the “Functional Non-painful” grade, then that specific movement must go to a breakout pattern to find the root cause of dysfunction. The “true cause of the dysfunction” can be a tissue extensibility dysfunction, a joint mobility dysfunction, or a stability/motor control issue (Goshtigian and Swanson, 2016; Fauntroy et al., 2019).

After the categorical scoring, subjects are assessed by the criterion checklist assigning an ordinal scale rating to each top-tier movement. A score of zero indicates perfect performance without compensation for all movements. A total score of 50 indicates failure of all criteria (Dolbeer et al., 2017; Kim and Do, 2021).

##### Layer 3: Detailed Breakout Assessments

Based on body pain diagram outcome, medical history and the SFMA test results, the operators were referred to one or more detailed test batteries carried out to further analyse lumbar, sacroiliac, cervical, lower limb, or upper limb dysfunctions. These additional tests enabled further identification of inefficient compensatory movement tendencies, muscular weaknesses, lack of motor control and stability, as well as lack of flexibility. This level of examination is only carried out for subjects for which more insights are needed and not routinely to everyone. As this third layer requires more technical devices such as isokinetic dynamometers, surface electromyography or pressure plates, these assessments are performed in laboratory conditions in the military hospital.

##### Layer 3A: Detailed Test Battery for Lower Back Dysfunctions

The test battery for lower back dysfunctions includes low load movement control tests like single and double knee extension in sitting, hip flexion in sitting, hip extension instance, and standing bow (Stevens et al., 2006; Van Damme et al., 2014), muscle extensibility tests (Lopes et al., 2021) and hip abductor strength test (Nadler et al., 2001). Additional isokinetic strength tests (trunk flexion and extension) and a 16-channel surface electromyography of the trunk muscles provided further insights if more high load and/or muscle endurance testing was needed.

##### Layer 3B: Detailed Test Battery for Cervical Dysfunctions

The test battery for cervical dysfunctions consisted of a broad spectrum of tests (Falla et al., 2004). Similar to all other body areas, the aim was to specifically address various impaired physiological functions, to propose a multimodal training regime (Blanpied et al., 2017).

Cervical ROM measurements for flexion, extension, lateral flexion, and rotation as well as proprioception were evaluated with Zebris, a 3 D-movement analyser (Zebris Medical GmbH, Isny, Germany). Mobility and proprioception were compared to normative data from a healthy military population within the same age and sex. The same applied for measurements of maximal isometric strength of neck flexors, extensors and lateral flexors with the David Back Dynamometer (Sihawong et al., 2011).

Thoracic posterior-anterior pressure tests were applied to investigate if there was thoracic involvement (Young et al., 2014). Observation of scapular (dys)function during arm anteflexion and abduction determined if cervico-scapulothoracic strengthening/stabilization exercises could be recommended (Helgadottir et al., 2010).

To address motor control of the cervical spine, segmental assessment was carried out by performing cranio-cervical flexion tests and deep sub-occipital extensor tests (Falla et al., 2004). Control of direction included low cervical flexion control-nodding and overhead arm lift, upper cervical flexion control-head forward lean and arm extension test, global rotation-sidebend control, neck global sidebend—rotation control, and upper cervical sidebend—upper neck tilt (Khosrokiani et al., 2018; Comerford and Mottram, 2019).

##### Layer 3C: Detailed Test Battery for Lower Limb Dysfunctions

The test battery for lower limb dysfunction comprised analytical mobility tests for hip, knee, ankle, and the first metatarsophalangeal joint. Hamstrings, hipflexor, hipadductor, hipabductor, and triceps surae extensibility tests were done as well, bearing in mind that flexibility plays an important role in reducing the risk for lower extremity musculoskeletal risk in special operation forces (Keenan et al., 2017).

Single leg stance static and dynamic control while reaching maximally with the other leg was evaluated during the Y Balance Test (Gribble and Hertel, 2003; Bressel et al., 2007). Additionally, tests for motor control during hip rotation, hip extension, heel walk, and stair descend followed for assessment of quality of movement (Herman et al., 2016; McGovern et al., 2018; Christopher et al., 2019).

Plantar pressure plate recording was performed [Footscan, RSscan Int, Paal (Belgium)]. It has been demonstrated to be useful to identify risk factors for overuse injury in a military population (Franklyn-Miller et al., 2014).

Maximal knee-extension and flexion strength (concentric and eccentric) were quantified using an isokinetic dynamometer. Knee-extension strength deficit is a known risk factor for musculoskeletal injury in operators (Barber et al., 1990).

##### Layer 3D: Detailed Test Battery for Upper Limb Dysfunctions

Research demonstrates that operators with a previous history of shoulder pain have less shoulder strength than uninjured operators (Parr et al., 2015). Therefore, concentric strength of internal and external shoulder rotators in 90° shoulder abduction was measured using an isokinetic dynamometer.

Further, scapula position (protraction, tilting) as well as scapular dynamic control during arm elevation and abduction was assessed. Anterior, posterior, multidirectional instability tests as well as load shift test were run and rotator cuff function of supraspinatus, infraspinatus and subscapularis was evaluated (Tennent et al., 2003; Lizzio et al., 2017). Additional detailed assessment of the elbow, wrist, and fingers was performed if needed (i.e., if operators had indicated issues regarding these locations in the screening questionnaire). Neurogenic testing, such as upper limb nerve provocation tests, was performed to collect detailed data subsequent to the clinical reasoning process of the therapists and concordant to the data collected in layer 2 (Nee et al., 2012). The sustained grip, pinch strength, and also range of motion in fingers and wrist was assessed using the Biometrics E-link (Biometrics Ltd, Newport, UK).

##### Conclusion Regarding Physiotherapy

In order to prevent and/or rehabilitate musculoskeletal problems in Special Forces operators, physical functioning was analyzed. 88% of all tested operators reported chronic or recurrent musculoskeletal symptoms, but it did not hold them from their professional occupations. A mean of three injuries or impairments are observed for each operator. One third are upper limb impairments (33%) which are more present in operators than in other military personnel. 31% of all injuries are located in the lower limb, 25% in the lumbar spine and 11% cervical spine impairments are observed. Therefore, over 80% of the screened operators were addressed to at least one of the layer 3 pathways.

As stated in the introduction, operators are, by definition, a small number of elite military personnel with a very high operational readiness level. This does not mean that those soldiers do not have to cope with injuries and consequences of past injuries. Over four out of five operators demonstrate musculoskeletal pain and/or dysfunctions impairing their physical readiness. Even without numerical comparisons, the management of a team of athletes will create similar conditions. In order to improve the readiness level, adding specialists in musculoskeletal rehabilitation and injury prevention in the day-to-day support to operators and athletes is paramount.

Feedback on test results immediately followed the screenings; corrective exercise programmes including stretching, muscle strengthening, motor control exercises, and/or manual therapy were provided to each operator individually. Exercise programs can both be monitored—and adapted—in the unit during consultations or remotely by the use of mobile applications. For the conception of this program, the Skill-Up (www.skill-up.com) and Physitrack (nl.physitrack.com) platforms were used to provide remote guidance and online exercise programs by the physiotherapist, in close cooperation with the PTI. The programs delivered by the physiotherapists have a focus on mobility, flexibility, motor control and strength upon which physical training programs can be further developed with an additional focus on conditioning, agility, endurance, balance, functional strength, speed, power… after an additional assessment done by the physical training instructors. This does also mean that if no major impairments are observed, an operator could skip the layers 1–3 and therefore immediately go to layer 4 (i.e., the PTI-managed level). The whole process covering the spectrum of assessment, rehabilitation, injury prevention, and customized training is detailed in Figure 2.

The ultimate goal of the functional musculoskeletal assessments is to create a framework for injury prevention, rehabilitation, and performance enhancement so that the operator could safely perform the movement demands of their military activities. Furthermore, considering the paramount importance for movement for this very physically active population, their ability to move pain free determines one of their main coping mechanisms (sports) and their overall quality of life.

#### Physical Training

As previously described, a major challenge in optimizing the physical readiness of SF operators resided in the combination of injury prevention and treatment on the one hand; and performance enhancement on the other hand. This challenge requires continuous communication between the physiotherapist and the physical trainer. The process is summarized in Figure 2, which also illustrates the practical application of one of the basic assumptions, being customization of training according to each operator's vulnerabilities. This need for interaction was reached by group meetings within the program team, and by sharing results of the clinical, the physical and the mental assessment. Indeed, the involvement of the performance psychologist in this physical assessment part also enabled the physiotherapist and the PTI to address issues in a way most suited to each individual, in terms of communication and leverage for behavioral change.

##### Physical Assessment: Rationale

Regarding physical training, the first step was to define a detailed physical performance assessment, which would be a key indicator for overall fitness; a reference to follow up training progress; and a blueprint of strengths and weaknesses to guide training. The choice of appropriate job-specific testing and training was determined by a literature review; conducting oral and written interviews with active operators; participant observations during some of the most physically demanding courses; and through international benchmarking and collaborations.

**Figure 3:**
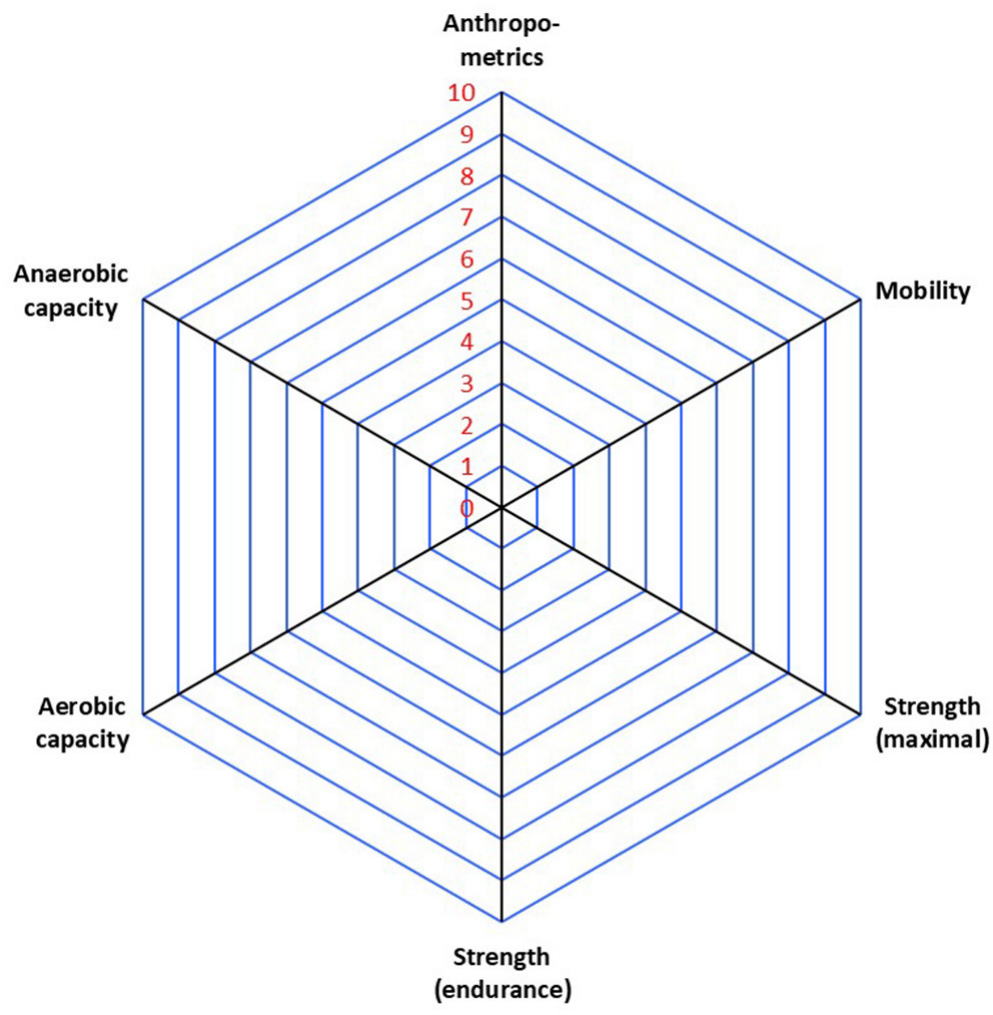
Overview of the SF operators' physical performance key determinants, as identified based on in-the-field observations, international benchmarking, and the available literature.

Pemrick (1999) evaluated the job demands of a comparable elite unit (i.e., the U.S. Army Rangers). Two specific missions carried out by the U.S. Rangers (i.e., a hostile raid and airfield seizure) were subjected to task analysis (Pemrick, 1999). This analysis highlighted three important physical components for the mission's success: aerobic-, anaerobic fitness and strength. Moreover, Eisinger et al. (2009) have performed a similar job analysis within the Austrian SOF community. Through further task analysis of operators' specific physical tasks (i.e., close combat, using explosives, parachuting, mountaineering, survival techniques, and shooting), it became clear that coordination and reaction speed are the most dominant physical components setting this population aside. Secondly, Eisinger et al. (2009) demonstrated, like Pemrick (1999), the importance of aerobic endurance, strength endurance and anaerobic endurance for SOF's work. Based on our participant observations, the international benchmarking and the available literature, we summarized the physical key components during training and missions in SOF operations in Figure 3.

Following the identification of these physical performance key components, specific tests had to be selected and combined in a physical fitness test battery. To optimize our choice of physical assessment tests, we evaluated the physical fitness test batteries previously used in a SOF context (Carlson and Jaenen, 2012; Sporiš et al., 2012; Solberg et al., 2015; Abt et al., 2016) and, considering the time-constraints, evaluated each test against a number of criteria to decide whether to include it or not. These inclusion criteria were: (i) The test had to measure in a valid and reliable way what is important to the nature of the military activity. Ideally, a test had to be as specific as possible to the job. However, because of the versatile nature of the array of tasks a SOF operator -and a team sports athlete- has to perform, there is an inevitable trade-off between standardization of the assessment and representativeness of the task. Whenever possible, we would favor validated tests in exercise science, in order to enable external comparisons. As emphasized by Vine et al. (2021), we strived for a balance between external validity and experimental control.; (ii) A test had to be practical, efficient, functional, convenient, and easy to perform; (iii) The test had to be as specific as possible, i.e., able to isolate and assess one physical key component at a time, to eventually be able to pinpoint someone's weakness(es) and avoid confounding results. Tests in which different physical key components were combined were thus avoided; (iv) In contrast to elite sports, there is generally very little time for individual testing within the SOF environment, due to the operational pressure. Whereas, training is seen as a “necessary evil” by both management and individual operators, testing, which is an investment to ensure training is targeted, is often not perceived as a justifiable time investment. Therefore, the tests had to be suitable for testing larger groups simultaneously in a short amount of time.

##### Physical Fitness Test Battery: Description

*Anthropometrics*. Body weight (BW) and body fat percentage (F%) were measured by using a bioelectrical impedance analysis (BIA, TANITA-BC-418 Segmental Body Composition Analyzer), with consideration of the measurement of weight up to the nearest 0.1 kg. These variables were measured because a prolonged intensive period of training can lead to an overall negative energy balance and low energy availability (Mullie et al., 2019; Rietjens et al., 2021). This low energy availability can lead to reductions in body weight (BW) and changes in body composition, which can impact both health and performance (Tassone and Baker, 2017). During an 8-week US Army Ranger Course, body weight losses averaged 9.4% with individuals losing to up to 17.5% of their body weight. A substantial loss of 3.6% of fat-free mass was observed during a 20-day training course in Finland. These body weight and body composition reductions and their impact on performance suggest the need for a better monitoring before and after field activities (Tassone and Baker, 2017).

*Mobility*. Whereas, an exhaustive assessment of mobility was already performed in the clinical assessment by physiotherapist, this “on-the-go” version allowed the PTI to follow-up in any location. A selection of four tests of the Functional Movement Screening (FMS) was applied, i.e., the deep squat, hurdle step, leg raise, and shoulder mobility (including the shoulder clearing test). This control check-up could be performed by the PTI, as results of all screenings were continuously shared between physiotherapists and physical trainers.

*Strength (Maximal)*. Four 3-repetition-maximum (3RM) tests were selected to assess maximal strength in different regions of the body where a large muscle mass is present and, in addition, to evaluate any imbalances in strength between specific body regions. The tests were leg press, bench press, vertical traction, and shoulder press.

*Strength (Endurance)*. Strength endurance was evaluated via two bodyweight exercises, the chin-up and the Biering-Sorensen test. The chin-up test is a dynamic strength endurance test in which the number of correct repetitions was used as outcome measure. A correct repetition included crossing the pull-up bar with the chin while keeping both legs together and without moving them forward, the hands had to be placed around the pull-up bar at shoulder width. In contrast, the Biering-Sorensen test is a static strength endurance test in which the outcome measure is the amount of time one can hold the correct position. In the Biering-Sorensen test one is secured to a horizontal table in the prone position. The table only supports the pelvis and legs. The test instruction is then as follows: maintain the horizontal position for as long as possible.

*Aerobic Capacity*. Regarding aerobic capacity, a 2-fold approach was followed: a “field testing” including a 2800-m run and a 16-km speed march, to allow for the PTI to perform the tests almost everywhere; and a laboratory testing to determine the actual VO_2_max.

The VO_2_max of each operator was measured by a maximal effort test on a treadmill. Despite this test being labor intensive and environmentally constrained by the availability of the equipment, the standardized outcome, if performed once a year for example, sustains the field assessment with more precise data. The maximal protocol started at 5.4 km/h, every 3 min the velocity was increased with 1.8 km/h, with a maximum of 23 km/h. Each 3-min stage, blood was drawn from the earlobe to evaluate blood lactate concentration. Gas exchange data with the operator's oxygen intake and carbon dioxide output measured was collected using an automated breath-by-breath system (Ergocard Clinical, Software Medisoft, Belgium). The relative VO_2_max was determined for maximal oxygen uptake in ml/kg/min and ml/min.

*Anaerobic Capacity (Speed)*. The Repeated High Intensity Test (RHIET) was applied, which consists of four repetitions of a 60 m sprint. Each 60 m sprint started 30 s after the previous run. This cycle continued until four sprints were completed, starting at 30 s, 1 min, 1.5 min after the start of the first sprint. A fatigue index was calculated by taking the average time of the first two trials and dividing it by the average time of the last two trials.

##### Performance and *Use* of the Test Battery

The time required to run this test battery was around 2 h for 20 people. Three to four instructors were needed to supervise the whole process.

**Figure 4:**
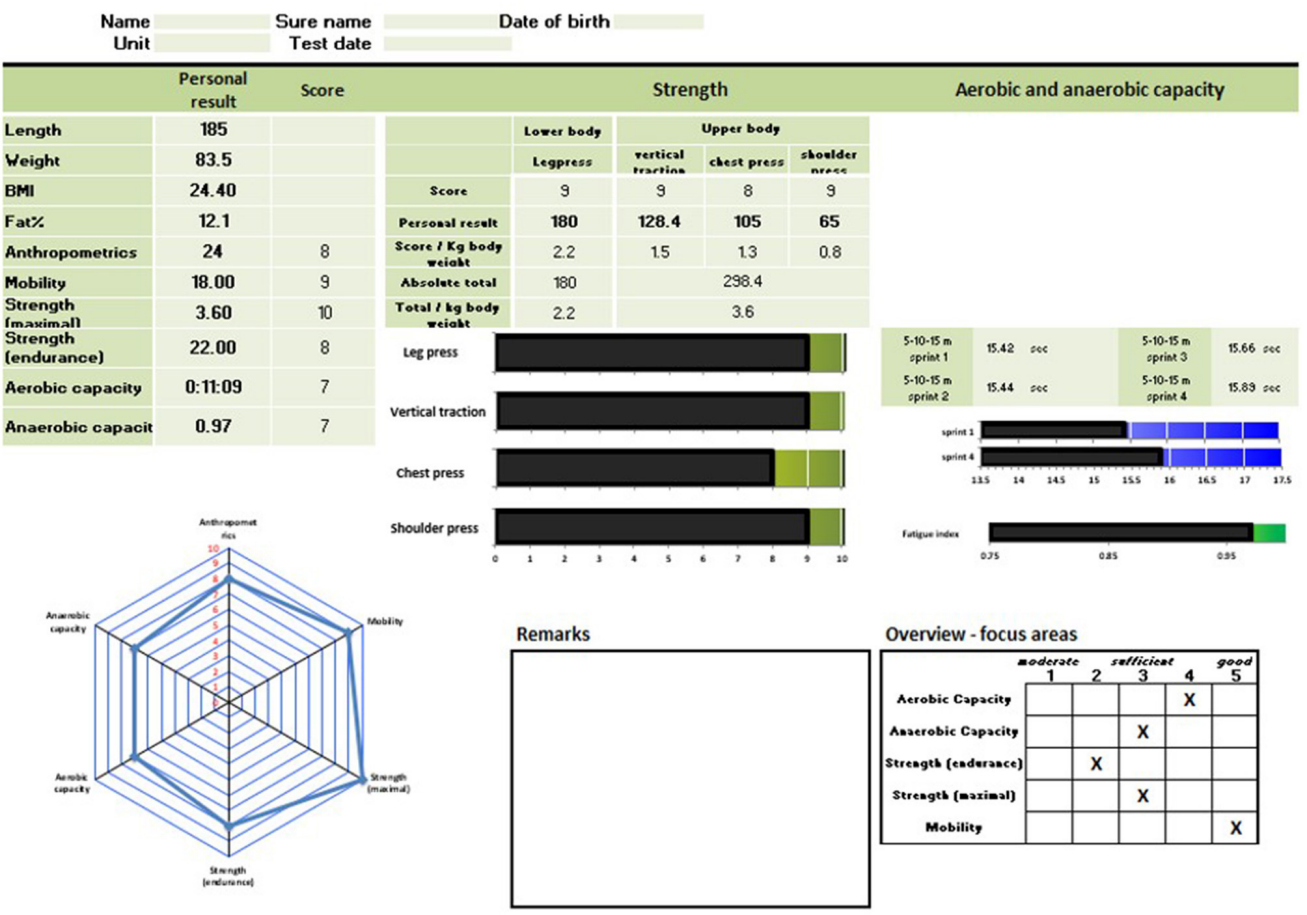
Example of the individual feedback of the physical assessment battery, according to the dimensions described above.

Commercial software is available to provide support with processing the test results. However, these are mainly made for (elite) sports and therefore not always suitable for our military arena, because of insufficient adaptability of the content. Furthermore, data security and ownership is often an issue, regarding server localizations and long-term guarantees. Lastly, use of the test battery on deployment requires a complete offline availability. For all these reasons we designed a simple MS Excel sheet to store and process the test data; and to produce an evaluation report, of which an example is provided in Figure 4.

The feedback of the test data was 2-fold. As an absolute number (score) for the individual; and as a percentile (10%) score in a spider diagram. In this diagram, the personal results were visualized in relation to their own peer group, being the other operators. When applied in the team training phase, this feedback allowed for the team to construct a “team report, ” identifying strengths and weaknesses of all team members, and already anticipating combat situations and role distributions. This stage will be further elaborated in the description of Step 4.

##### Individualized Training Program

Based on the results of the test battery, which were discussed within the multidisciplinary project team, the goal was to address the observed weaknesses and maintain the individual's strengths. Physical training was provided to the tested SF operators in two ways: (i) via a team training in which the weaknesses of the group in general were specifically addressed, and which was also the first stage in the team training that will be described in the specific section (Step 4); and (ii) via an individualized training that could be performed at customized times. Both group trainings and individualized training programs were developed in close collaboration with the physiotherapist and with the operators themselves, during the individual consultations. Individual feedback moments were organized with each tested SF operator. To aim for a certain level of periodization, and to determine when intensive training blocks could be performed, the PTI coordinated with the team leader, to discuss the macrocyclus of the upcoming work-related activities (exercises, deployments…). The tactical planning was thus taken into account in developing individualized training programs, and in the decision on when to focus training on addressing a specific weakness or on maintaining a specific strength, or on recovery to prepare for particularly intensive operational demands.

A specific advantage of individualizing training programs was also that it provided the ability to be more focused and therefore more time-efficient. Due to operators' operational readiness and workload, it was difficult to plan for yet another training program in their already busy schedule, an issue that was already acknowledged in previous literature (Christensen et al., 2008). Furthermore, if training is possible, their deployments do not always allow for optimal training conditions (e.g., sufficient time, sleep deprivation, lack of food intake, or the adequate facilities), which can alter their operational readiness and result in higher injury rates (Sharp et al., 2008). The high level of customization; the mutual trust relationship between the operators, the PTI and their dedicated physiotherapist; and the availability of the experts for reach back consultation during exercises and deployments were key features in the adherence to the training program.

##### Nutrition and Hydration

Elite forces preparation involves such metabolic demands, that daily training may require up to 6,000 kcal/day. Such high energy needs pose a major challenge to maintaining the energy balance over a longer period of time. Risks such as insufficient energy intake from carbohydrates, dehydration due to low fluid intake and the intake of poor-quality nutrition (a lot of energy, but insufficient nutrients) are lurking and can eventually lead to weight loss through muscle breakdown and significant decreases in mental and cognitive performance. In 2019, Mullie et al. published a study on energy expenditure and availability within the Belgian Special Forces qualification course. During four consecutive days, candidates undertaking the Q-course (i.e., the qualification course to become a SOF operator) were assessed. Through measuring hydration, recording physical activity and registering the intake of nutrients, important nutritional deficiencies were identified. With only 17 kcal per kg fat free mass (FFM) per day, mean energy availability was far below the recommended 40 kcal per kg FFM per day to perform in optimal conditions, i.e., without a decline in essential physiological processes (Loucks et al., 2011). When prolonged, low energy availability can lead to adverse physiological and psychological effects and impair performance and health (Logue et al., 2018).

Tackling nutrition in a Defense environment is complex, for the purchase and procurement decision is usually made higher up than at the scale of the unit. Ideally, quality and quantity of available food should be improved, by taking into account composition (macro and micronutrients), portion size, timing of offering, taste, and presentation. In the context of SOF, the main challenge is not to determine precisely how to scientifically investigate what optimum type of nutrition should be offered. Several comprehensive sources already exist that cover the topic (e.g., Deuster et al., 2017; Rietjens et al., 2021). The main issue is a practical one, which can be summarized as timing (of food intake) and availability (of food of sufficient quality in sufficient quantity).

Both Deuster et al. (2017) and Cole et al. (2018) thus acknowledge the need to educate the end-user (i.e., the operator) as the first step in improving nutrition in a SOF population. Cole et al. (2018) showed that education resulted in diet quality improvements and thus demonstrated this to be feasible to be implemented in a Special Operations Forces Human Performance Program. Since the frame of our project did not allow for a direct intervention regarding procurement or mess organization in the barracks, this was indeed the only level we could target. The implementation of this nutrition education will be described in the “Implementation section, ” paragraph Step 4: Implementation in a Pilot Project: A One-Year Follow-up of One SOF Team.

#### Performance Psychology

As discussed in the introduction, we aimed to overcome several dualisms in the performance psychology approach. We set out to evaluate the holistic subjective experience of the operator from a systemic point of view. As physical performance is of paramount importance for the professional demands of our population, it was important to encompass this aspect in the subjective evaluation. Furthermore, overall well-being at work and at home were also considered relevant. SOF operators and elite athlete both commit completely to their profession, hence the impossibility to compartmentalize their support, which is emphasized by Barry and De Vries (2019): “USASOC Strategy 2035 Campaign Plan codified the need for ARSOF to improve human/spiritual performance, behavioral health, social readiness, and resilience.” As we described in the introduction and method, psychology actually knows several sub-disciplines, and the aspect of performance psychology was the product of a constant interaction within the program design team. As we discussed in the “Method” section, and considering the influence for our other variables, we have included sleep in this layer as well. Sleep and fatigue management were thus a vector of both assessments and interventions. Whereas, this domain has widely been acknowledged as a major determinant of performance in other areas, such as aviation (for a review, see Caldwell and Caldwell, 2005), it seems this subject only gained momentum in the tactical army population in the last decade (e.g., Troxel et al., 2015). Furthermore, based on our observations, this was an important area of concern. The holistic and systemic approach we applied does not only mean to add several perspectives, but also to evaluate how they influence one another on the one hand and how the individual's well-being influences the team and vice versa. Achieving this required an enhanced targeted communication toward this goal, both within the multidisciplinary project team on the one hand, and within the pilot project SOF team on the other hand. Both teams thus managed to enhance their situational awareness to the “bigger picture” and increase insight and reflection both on the process and on the content.

As described in the “Method” section, the first step was to select the relevant individual assessment to work with. We thus layered the experiential assessment in three layers: the first layer was the individual screening and mapping based on psychometry; the second layer was the customization and individualized feedback step; the third layer was the integration at team level, comprising the psychoeducation approach to the team to empower self-regulation.

##### Layer 1 = Overall Screening and Mapping Based on Psychometry

Two types of psychometry tools were used. The first one encompasses all the “trait”-like variables, hence considered to be stable within one individual. The second one covered the “state”-like variable, meaning situationally sensitive variables, which vary with changing resources and demands (e.g., sleep deprivation, family issue, physical fatigue etc).

*Psychometry: Trait Assessments*. A “trait”-like feature measure only needs to be taken once, to provide a profile of the individual. Our choice of individual psychometric tools to measure “trait” like variables was guided by three criteria: (i) the necessity to rely on validated instruments, in order to be able to interpret results against standardized norms and compare to other investigations; (ii) what we called the “bottom-up feed”: the information we received from the unit, regarding the “ideal” profile of an operator in their perception, based on decades of operational experience, coupled to our year-round observation of courses and deployment integrated to the operational detachment (as detailed in the Method section), which allowed us to make an informed choice regarding target variables related to real-life performance; (iii) the available literature regarding psychometric investigations related to performance prediction in SOF. We chose to measure personality dimensions, intelligence-fluid, crystallized and emotional- and sleep traits. The following section summarizes the rationale for these variables, as well as which instruments were implemented. All testing was conducted by a trained clinical neuropsychologist and in the native language of the operators. These language variations are not reported in the current paper.

The NEO-PI-R was used to have an overall assessment of personality (Costa and McCrae, 2008). As we described earlier, operators thrive in environments that are actually extremely stressful. Several moderators, such as hardiness, can sustain this thriving (Bartone, 1999). Based on the existing literature, we opted to measure several trait-like aspects of psychological fitness, i.e., hardiness, risk-taking, and trait-anxiety.

Hardiness is a fairly stable personality trait, which has been shown to predict a successful outcome of the qualification course (Bartone et al., 2008; Hystad et al., 2010; Lo Bue, 2015). Hanton et al. (2013) examined the interaction between hardiness and anxiety and found that people who scored high on hardiness also tended to have lower levels of anxiety. Hardiness was evaluated using the Hardiness questionnaire developed by Lo Bue (2015). This questionnaire consists of 40 affirmative sentences. 24 items are “positively” connoted and measure commitment (7), control (11), and challenge (6). These three components constitute the construct of dispositional resilience. Sixteen items are “negatively” connoted and measure alienation (negative commitment), powerlessness (negative control) and rigidity (negative challenge). These three negative components constitute the construct of dispositional vulnerability. These two composite scores allow to calculate the total hardiness score.

The eagerness to be exposed to danger, sensation-seeking and risk-taking tendencies are beneficial traits for war fighters (e.g., Momen et al., 2010). Risk-taking has shown to correlate significantly with successful completion of the SOF training (Pleban et al., 1989). Unsurprisingly, sensation-seeking is closely linked with risk-taking (Momen et al., 2010). Risk-taking is inherent to any SOF task and thus, according to Momen et al. (2010), the ideal war fighter is a ‘deliberative sensation-seeker' (Momen et al., 2010). Risk-taking was assessed using the revised Domain-Specific Risk Taking (DOSPERT) Scale (Blais and Weber, 2006). This 30-item scale assesses behavioral intentions—or the likelihood that respondents will engage in risky activities—from five areas of life (ethical, financial, health/safety, social, and leisure risks). Higher scores indicate greater risk taking in the subscale area. A second scale of 30 items assesses the perceived degree of risk of each activity/behavior. The combination of both subscales is interesting, especially in this population, to evaluate whether “risky” behavior is indeed perceived as “risky.”

Trait anxiety was assessed with the trait scale of the State-Trait Anxiety Inventory (STAI-T; Spielberger et al., 1983). This questionnaire has 20 items including both anxiety-dependent items, e.g., “I am too worried about something that doesn't really matter”; as well as anxiety-independent items, e.g., “I am a stable person.” The total score ranges from 20 to 80; the higher the score, the higher the anxiety trait in the individual.

Operators must be able to receive, understand, memorize, and integrate large amounts of complex information (e.g., verbally receive information, procedures, or sophisticated materials) in a short period of time, and in constantly changing settings (e.g., Picano et al., 2017; Farina et al., 2019). Intelligence was evaluated by the Wechsler Adult Intelligence Scale-Fourth Edition (WAIS-IV; Wechsler, 2008). The ten core subtests are required to calculate the Full-Scale IQ (FSIQ) and the four following indexes: (i) Verbal Comprehension Index (VCI), (ii) Perceptual Reasoning Index (PRI), (iii) Working Memory Index (WMI), and (iv) Processing Speed Index (PSI).

Despite the lack of available literature in military populations regarding emotional intelligence and performance, it has recently gained momentum in sports science (for a review, see Laborde et al., 2016). Emotional intelligence (EI) can be defined as emotional literacy: “the ability to perceive and express emotion, assimilate emotion in thought, understand and reason with emotion, and regulate emotion in the self and others” (Mayer et al., 2000, p. 396; see also Mayer and Salovey, 1997). Layman popularization publications link the concept to motivation, empathy, communication and interpersonal skills (e.g., Goleman, 1995). According to the standard definition, EI consists of four attributes: (i) the ability to perceive, assess, and express emotions quickly, (ii) the ability to recognize and generate the feelings that facilitate thinking, (iii) the ability to understand emotions and knowledge about emotions, (iv) the ability to manage emotions in order to improve emotional and intellectual development (Salovey and Mayer, 1990). Considering the body of research linking this ability to performance in various domains (e.g., Vaughan et al., 2021, linking trait EI to working memory with a growing weight of the relationship with elite status of the athletes), and considering the fact that one of our core assumptions for the current program was to target the team as the basic unit for performance, and thus to have a reliable measure of interpersonal skills related to the quality of the team experience, we included EI in our measurements. Many different EI assessment questionnaires exist, and we decided to choose the one having produced the most research results so far, also being the oldest, being the Bar-On (1997).

Regarding sleep and fatigue, current research also indicates a growing acknowledgment of stable interindividual differences influencing sleep need, vulnerability of performance to sleep loss, and circadian set-up (e.g., Tucker et al., 2007). Two of these clearly identified traits are the sleep need (i.e., normal duration of sleep during one night) and the chronotype (i.e., the circadian preference, as in morningness or eveningness). Regarding the sleep need, a 2-weeks sleep diary has been the easiest method for determining this for decades (Monk et al., 1994). Regarding the chronotype, several questionnaires exist. A recent tool, the Oginska Chronotype Questionnaire (Ogińska, 2011; Oginska et al., 2017) allows for a shorter version mapping both morningness-eveningness, but also diurnal amplitude, that is the range of change in arousal and responsiveness (which has been linked to stress responses) throughout the day. The questionnaire consists of 14 items. Two dimensions of the chronotype are assessed using this questionnaire: the morning-vesperality scale (i.e., the so-called morningness-eveningness, ME; 8 items) and the subjective amplitude scale (i.e., DI; 6 items). The higher the score on the ME-scale, the more it reflects a tendency to be more active in the evening. The subjective amplitude investigates the subjective sense of distinction of daily changes (i.e., the amplitude or range of diurnal fluctuations). The amplitude would reflect the power of the human circadian system (Aschoff and Pohl, 1978). The higher the score on this scale, the stronger the subjective distinction in diurnal variations. Amplitude characteristics appear to be considered an important component of circadian rhythms, particularly in the workplace where it could predict an individual's tolerance to shift work or individual jet lag. Considering the ease of use of this questionnaire, as well as the additional information of amplitude which might be relevant to operators, especially in situations of jet lag, we thus chose this instrument. The different instruments to measure trait variables are summarized in Table 2.

**Table 2:** Summary of the psychometry tools used for mapping of the selected trait variables.

**Variable**	**Instrument**	**Source**	**Administration duration**	**Scoring duration**
Personality	NEO-PI-R	Costa and McCrae (2008)	50 min	Automated
Anxiety	STAI-T	Spielberger (1983)	7 min	Automated
Hardiness	Lo Bue	Lo Bue (2015)	15 min	Automated
Risk-taking	DOSPERT	Blais and Weber (2006)	20 min	10 min (manual comparison of both subscales needed)
Emotional intelligence	Bar-On EQi	Bar-On (1997)	30 min	Automated
Intelligence	WAIS IV	Wechsler (2008)	2 h	30 min
Chronotype	Ogińska	Ogińska (2011)	7 min	Automated
Sleep need	Sleep diary	Monk et al. (1994)	5 min daily for ~2 weeks	30 min

*The durations to score do not include the initial time investment needed to design a scoring interface*.

*Psychometry: State Assessments*. The second type of psychometry tools maps state-like variables, likely to vary in different situations, and where the outcome or score might provide an indication regarding the resource use of the individual. Contrary to the trait-like variables, these indicators might be used in a dynamic setting, and be self-managed and monitored by the operators, following the appropriate psychoeducation. Again, our choice was guided by several criteria: (i) the ease of use and interpretation, to be applied without an expert, hence the possibility to fully automate and self-administer the measures and make the operator self-sufficient in its use; (ii) a duration of administration that is short enough to allow for an administration “on the go”; (iii) a well-documented and validated sensitivity to situational variables likely to affect performance in our population. The state questionnaires (i.e., profile of mood scale, state anxiety scale, sleep quality as well as subjective level of mental fatigue, physical fatigue, stress and sleepiness) specifically assessed the psychological state of the participants at different moments in time (baseline measures, during courses and deployments) to provide individuals with a referential framework. Table 3 provides an overview of these different instruments. The variables chosen were anxiety (Spielberger STAI S-subscale), overall mood state and sleep quality.

**Table 3:** Summary of the psychometry tools used for the psychological mapping of selected state variables to allow for self-monitoring.

**Variable**	**Instrument**	**Source**	**Administration duration**	**Administration frequency**	**Scoring duration**
Anxiety	STAI-S	Spielberger (1983)	7 min	On demand	Automated through an online interface
Fatigue	VAS	Frey (2018)	10 s	On demand	
Sleepiness	VAS	Frey (2018)	10 s	On demand	
Psychomotor response speed	PVT	Dinges and Powell (1985)	10 min	On demand, circadian influence on result	
Sleep quality	PSQI	Buysse et al. (1989)	10 min	Monthly	
Mood	POMS	Curran et al. (1995)	7 min	On demand	

*The “on demand” description of administration frequency indicates the possibility for operators to take the measure whenever they deemed appropriate or necessary*.

Regarding overall mood, the Profile Of Mood States (POMS) has become the most widely used instrument in applied research. The Profile of Mood States (POMS) questionnaire can be a key instrument in reporting the negative and positive mood states changes during exhaustive periods of training in athletes (Meeusen et al., 2013). The 32-items POMS has five subscales: tension-anxiety (POMS-T), depression-dejection (POMS-D), anger-hostility (POMS-A), fatigue-inertia (POMS-F), and vigor-activity (POMS-V). For athletes, the “Iceberg Profile” has been acknowledged as the healthy norm. This is a representation of POMS scores, with scores below population average on the subscales fatigue, depression, tension and anger, and a sky-high “top of the iceberg” score on the subscale vigor. This positive visual profile is typical and very common for well-trained athletes (Vrijkotte et al., 2016). The Iceberg Profile of SF operators and their candidates has already been compared to elite athletes (Johnson et al., 2019).

Experiential sampling is a unique way to gain insight in how a person perceives mood, bodily sensations, feelings, and resource allocation during a given activity. In order to give operators insight in this quantification, we taught them to use Visual Analog Scales to log “Mental Fatigue, ” “Physical Fatigue, ” “Sleepiness, ” and “Stress.” The Visual Analog Scale (VAS) we used is a 100 mm horizontal rating scale without numbers, where participants mark a point that indicates the intensity of the subjective phenomenon. The ends are extreme limits of the parameter to be measured, in this case the extremities vary between the limits “not fatigued/stressed/sleepy at all” on the left side and “extremely exhausted/stressed/sleepy” on the right side of the line. The VAS has shown to be a fast and reliable instrument in this population (Vrijkotte et al., 2018).

Regarding sleep, we also included a monthly Pittsburgh Sleep Quality Inventory (PSQI), which has been the clinical gold standard for decades for screening and follow-up of sleep quality (Buysse et al., 1989). The PSQI consists of 21 questions. Each question measures a specific area in which sleep problems might occur. Seven components are assessed; and their associated questions are as follows: Component 1, subjective sleep quality-question 9; Component 2, sleep latency-questions 2 and 5a; Component 3, sleep duration-question 4; Component 4, habitual sleep efficiency-questions 1, 3, and 4; Component 5, sleep disturbances-questions 5b through 5j; Component 6, use of sleep-promoting medications-question 6; Component 7, daytime dysfunction-questions 7 and 8. The formulation of the questions targets variations and state over the last month.

Regarding anxiety, since the original instrument designed by Spielberger specifically differentiated between trait and state anxiety, the “State” subscale was used to include in the situational variables.

##### Layer 2 = Individual Feedback Based on the Results of the Assessment

The core of this step was the customization. The information collected in “Layer 1” was fed back in individual interviews with the operators. A clinical systemic interview was conducted with each of them by a trained systemic psychotherapist, as well as an interview with a performance psychologist, as well as a joint feedback interview with both. It is unusual in psychology to have both disciplines work closely together, yet this seemed a necessity considering our basic requirements formulated earlier. During these interviews, individual strengths and weaknesses were identified and discussed. Where necessary, further investigations and potential interventions were discussed. An example of such a referral was the identification of a sleep pathology, where the referral to a clinical sleep specialist allowed for the identification of an obstructive sleep apnoea syndrome, which could be surgically treated. Another example of the operational use of this personalized feedback was the choice of team specialty by one of the younger operators, who had initially been directed to the “sharpshooter/sniper” track, but who expressed a wish to change this based on his individual cognitive feedback.

Regarding the clinical interview, as this was constructed as a systemic “intake, ” it explored personal relationship and family life as well, and if necessary or if the operator expressed the need for it, family interventions were also scheduled. This is in line with one of the objectives stated in the Method section, regarding the support of partner relationship, being one of the coping mechanisms of the operators.

A paramount feature of this stage was the consultation within the project design team, to be able to integrate feedback and interventions from the different areas of expertise (physical training, physiotherapy, medical consultations, and psychological assessments and interviews). Between the interviews and the interventions, each operator's case was discussed within the multidisciplinary team, in order to ensure the full situational awareness of each expert regarding the most appropriate care for this operator.

Furthermore, the steps of the next layer, being the integration in the team, were discussed at this stage within each individual consultation, to allow each individual sufficient time to determine his boundaries regarding individual and team feedback. This also paved the way for this first explicit intervention in team dynamics with experts from “outside” the operators' community. As indicated by our initial survey regarding psychological support (Huret, 2018), which we summarized in the “Methods” section, the trust relationship that had been forged by the repeated participant observations was of paramount importance to obtain buy-in from the individual operators at this stage.

The main message from this layer on was the responsabilisation of the individual, to reach a co-creation of the guidance and support process with the professionals, consistent with the therapeutic assumptions in systemic psychology (Jorgensen, 1989; McTaggart, 1991; McIntyre, 2007; Gergen, 2008; Spradley, 2016).

##### Layer 3 = Team Feedback and Interventions

As Hollnagel (1998) wrote more than two decades ago: “in the study of human performance the definition or specification of what one should measure is undoubtedly the most important problem, whether for individual or crew performance. Measurements must meet three essential requirements: (i) they must be possible; (ii) they must be reliable; and (iii) they must be meaningful or valid. Very few of the measurements that are used in practice meet all three requirements.” This has remained an issue in the field of team performance. We posit that the type of test matters, however, one variable often overlooked when working with assessment results is the quality of the expert who provides the feedback and thus uses the information. In this programme, all psychometry results were discussed, fed back and integrated in team workshop guided by a combination of a trained clinical psychologists, a neuropsychologist, a MD and a performance psychologist.

Regarding personality and team interactions, we based our team feedback and interventions on the NEO-PI-R and the EQi results. Considering the fact that the operators had been exposed to the Myers-Briggs Type Inventory (Myers, 1962) in a former international training, and wished to build further on these notions, we coupled the MBTI typology to the feedback from the NEO-PI-R, based on McCrae and Costa's work in the field McCrae and Costa (1989) and the EQi. This situation actually exemplifies the experience of a lot of psychology professionals with the MBTI. As stated by Stein and Swan (2019), “Despite its immense popularity and impressive longevity, the Myers-Briggs Type Indicator (MBTI) has existed in a parallel universe to social and personality psychology.” These authors provide a rigorous analysis on the theoretical assumptions behind the MBTI as well as a review of available evidence confirming/infirming the use of the test. They also explain in detail why it might be counterproductive, given the popularity of the instrument in a general public, to try and convince people of its inherent flaws. They describe a potential pragmatic use of the test, as a kind of “door-opener” to awareness regarding one's own functioning, which, as they state, is what is also advocated by the company selling the instrument. Indeed, as identified by Stein and Swan, “the MBTI is sold not necessarily on its theoretical rigor but on its ability to help its users (Stein and Swan, 2019).” We thus followed these authors pragmatic vision, and actually used the MBTI as a simplifying language transition between other tested dimensions and our feedback.

Considering the small size and very equalitarian structure of the team we were working with, we included a “third-person” assessment rather than a 360° feedback. This encompassed filling in an MBTI as each and every other team members, in order to qualify the difference between self-perception and perception by others. This relates to the concept of social desirability and authenticity, which we deemed worthy of more investigation, considering the importance of team dynamics as a coping mechanism in our initial survey.

Usually, in the context of assessment, social desirability is discussed as a bias threatening the reliability of questionnaires assessment. According to Paulhus (1984), social desirability could be viewed in terms of either self-deception (SD), or impression management (IM). IM is a conscious process in which participants intentionally dissimulate information to create a socially desirable image (Wrangham, 1999). Therefore, Paulhus (1984) recommends controlling IM in personality measures as it may represent a conscious bias (Burns and Christiansen, 2006). In contrast, SD refers to instances when respondents actually believe their positive self-reports (i.e., positive illusions). In non-military contexts, positive illusions appear to enhance performance by deflecting attention from anxiety, pain, and fatigue, both among groups and individuals (Wrangham, 1999). SD reveals to some degree how respondents subconsciously alter their answers to protect their self-esteem (Bobbio and Manganelli, 2011). Knowing the importance of positive self-esteem to effectively cope with worksite adversity in any demanding situation (Folkman and Moskowitz, 2004), SD may also underlie success in military context (Wrangham, 1999). Since the success of this team intervention was based on the willingness to share authentically, we included social desirability in the assessment, in order to make participants aware of their dispositions regarding this aspect of their behavior. The social desirability was measured using the Balanced Inventory of Desirable Responding (BIDR; Paulhus, 1984, 1991). The BIDR includes 40 statements and scores can range from 20 to 140, with the highest scores reflecting the highest levels of either self-deception or impression management.

As mutual trust among team members is consistently cited by operators as one of the core features of the “SOF mindset, ” we targeted this dimension as the cornerstone of the team intervention in a systemic framework. Indeed, coupling the results of individual feedbacks (from the NEO-PI-R, the EQi, and the MBTI) to the results of the “third-person” assessments and the BIDR test results allowed for a workshop covering authenticity and differences in interpersonal functioning. Each team member identifyed defining features of his team mates, as well as differences and common denominators in their individual functioning regarding personal preferences in problem solving, decision making and communication. This took place over the course of 1 week, in three sessions lasting each for 3 h, in order to allow for a sufficient maturation of the information and the feedback, to ensure a common mental model of the team dynamics as the end goal. The evolution of team dynamics over the course of these three sessions was noteworthy, as it served the team reinforcing purpose. The team started as a recipient of experts' feedback during the first session, to move to autonomous communication handling the vocabulary of teamwork, communication, and collaborative decision making in the last session. In this way individual feedback moments of individual experts were exchanged on a team level in function of the team and these team dynamics nurtured the individual motivation and performance goals. This was the foundation for the implementation of the team training, which we describe in the next section.

### Step 4: Implementation in a Pilot Project: A One-Year Follow-Up of One SOF Team

#### The Multidisciplinarity of the Team Training

In order to fulfill one of our core objectives, being a holistic multidisciplinary approach, the specialists' program had to be woven together, in a way where each professional would understand and buy-in to the interventions from the other, and identify potential synergies. This required several coordination meetings, and the opportunity for each specialist to learn about the assessment and interventions of the other fields. The coordination meetings were also the opportunity to schedule our pilot project implementation. Considering the high operational tempo for operators, a modular approach of 4 weeks over 1 year was chosen, which would be completed with *ad-hoc* interventions on deployment, either with an expert deployed as part of the team, or with a reach back capacity.

Prior to the start of this follow-up, each specialist educated the other team members regarding his/her approach (i.e., the content of the previous sections of this paper), explaining the “what, ” the “why, ” and the “how” of the individual assessment, feedback and interventions. Furthermore, during each of the 4 weeks, all the experts freed their schedule to be completely available, and whenever possible to partake in all the activities of the program. This served a 2-fold purpose: on the one hand it ensured an in-depth knowledge and understanding of the full program in order to identify all the possible synergies and leverages to better coach the operators in each domain; on the other hand, it demonstrated the practical implication of each expert to the participating operators, thereby demonstrating that the co-creation of this program was not a hollow buzzword, but a practical reality. The sum of the preparatory work and the availability during those weeks meant a significant additional workload for the involved experts, but also a hugely rewarding experience.

One component of the program which we have not described yet, but which was essential to ensure the eventual autonomy of the team, and the impact of the feedback in the different areas, is an educational component. In the SOF community, the need for training operators on the weapon systems they use would never be questioned, however there are virtually no resources devoted to educating operators in the function of their own weapon system—their brain and body. Practical training in weapons and tactics (for example) involve theoretical and practical applications leading up to real time, full mission profile activities. We chose this same approach to a basic introduction in how humans work as a system, on the individual and team level. The summarized content of this training is described in the following section. The different layers of the previously described areas of expertise are woven together in an integrative modular approach, where we start with individual assessments and education and work up toward team autonomy.

#### From Individual to Team Training: How to Make the Whole Greater Than the Sum of the Parts

Section Layer 3 = Team Feedback and Interventions “Team feedback and interventions” already provided a first glimpse in the process of transfer between the individual assessment, feedback and interventions, and the team level. This was a process that was new for both many of the involved experts and for the operators. Indeed, in the respective areas of clinical medicine, physiotherapy, physical training, and performance psychology, the focus is always on the individual organism: its strengths, weaknesses, and the customization of the necessary interventions for that particular person. The only expert used to work with “networks” of individuals was the systemic psychotherapist. However, one of the basic tenets of our program design was to overcome the duality between individual and team training. Traditionally, operators are selected and trained to aim to be the best. Hence, moving away from this purely individualistic perspective of performance management, while keeping personal standards at a level of excellence was a shift that required some mindset adjustment, moving away from a maximized development of individuals to a balanced development to maximize team performance.

This adjustment was the product of the whole process of this training, however, three interventions explicitly targeted this effect. The first one was an illustrated analogy between the memoir of Chris Hadfield on his career as an astronaut (Hadfield, 2015) and the career of an operator. Hadfield very clearly describes the shift in his mindset between being a competitive, individualistic fighter pilot to being a crew member from a space crew, realizing that his survival chances in space and his possibility to attain execellence depended on the quality of crew performance rather than on his own. Hadfield uses the phrases “how to be a zero” to characterize this mindset shift: how he moved away from trying to be the noticeable best in any system he was involved in, to trying to be a most fluid and efficient cog in an extremely complex machine. The second one was the team intervention described earlier, where team members received and discussed feedback regarding personality assessments and team functioning, during a workshop targeting trust in the team. The third one was a classroom workshop examining real cases of performance assessment and human error in previous courses and deployments, based on our participant observations. These included performance scores for physical or tactical challenges in courses, or elements from after action reviews from deployments. This stage allowed to demonstrate that individuals that might have seemed like “high performers” were actually depending on the system (team, unit) in which they functioned in order to deliver real-life high performance. All these interventions are summarized in the “Implementation blueprint” section further on. As the team was a pivotal element in this aspect of the process, the fact that they further applied these notions in team activities outside our human performance program was paramount to its success, and was to us a clear indicator of the adequacy of the chosen approach.

For the expert team, the process was guided by the theoretical framework of system theory. As stated in the Method, our program design was a non-hierarchical co-creation between the experts and the actual client, and in this particular stage, the focus was intentionally slowly shifted from the individual client to the team as client. This took place over the course of the 1-year follow-up, with a strong shift as of the second week of the program, where all the individual assessment and feedback had taken place during the first week, and where this second week saw the emergence of “the team” as the client, rather than each individual.

#### Implementation Blueprint: The Kitchen Recipe

We thus conceived the program as a modular build-up around six periods throughout the year: 4 weeks at the unit, hence neither deployed nor in training; and two deployment periods. The four weeks at the unit are the only ones throughout a whole year where a team is together in a “normal” work environment, they are called the “administration and logistics” weeks. The fact that there are only four of those weeks in a year underscores the operational pressure we have mentioned throughout the current paper. The two deployment periods were one mountain training of 3 weeks, where the PTI was the embedded expert; and one operational deployment of 3 months, where the MD was the embedded expert for 1 month. The content was built starting from individual assessment and education to feedback and insight, allowing for practical workshops empowering autonomy and targeted intervention in function of needs. As mentioned before, the focus shifted from the individual to the team as soon as the second week. Table 4 provides an overview of the schedule and organization for the team members (hence not taking into account the preparatory work and coordination between the experts). As the year went by, the process evolved from an expert-giving-counsel model to a true co-development with the team, based on the developing insights and experience.

**Table 4:** Implementation blueprint.

**Administration and logistics weeks**			
BLOCK 1	1 week (Jan)	Education (team classroom sessions)	1. Introduction to the program and goalsetting (1 h) 2. Exercise physiology and training principles (4 h) 3. Information processing and learning processes (4 h)
		Individual assessment	1. Initial medical interview 2. Psychometry tools 3. Individual intake interview with the clinical psychologist 4. VO2 max testing at the sports physiology laboratory 5. Individual physiotherapy screening: questionnaire and consultations
		Team intervention	First team training session with PTI to illustrate training principles (half day)
BLOCK 2	1 week (Apr)	Education (team classroom sessions)	1. Nutrition basics (4 hrs) 2. Communication and team cognition (2 h) 3. Sleep and fatigue management for optimal performance (2 hrs)
		Individual assessments	1. Full physical assessment with PTI 2. Repeat sleep aspects of psychometry
		Individual intervention/Feedback	Individual consultation with physiotherapist and PTI to discuss customized training program based on the assessments of block 1.
		Workshop/Practical exercise	1. Nutrition: analysis of the different types of field rations used by the unit 2. Exercise on determination of metabolic needs in function of different types of settings and activities (based on real exercises/deployments) 3. Sleep and fatigue: scheduling examples based on observational data from the mission of the previous year
		Feedback	Individual interview with the psychologists regarding the psychometry results from block 1.
		Team intervention	1. Introducing the concept of team performance management and the team assessments 2. Group workshops around personality types, behavioral preferences, and team dynamics
BLOCK 3	1 week (Jun)	Individual intervention	1. Individual follow-up with physiotherapist and PTI on customized training program 2. Individual pre-deployment interview with clinical psychologist
		Team intervention/workshop	1. How to implement the Human Performance Program on deployment. 2. Team cognition, performance, and human error: how to reframe error analyses (with real-cases examples). 3. Team training session with PTI
BLOCK 4	1 week (Dec)	Individual assessment	Repeat of the full physical assessment to evaluate impact of deployment.
		Individual intervention	Follow-up with physiotherapist and PTI on customized training program. Follow-up consultation with ad hoc experts based on individual needs.
		Team intervention/ workshop	1. Debriefing on human performance aspects on deployment: physical activity, nutrition, sleep and fatigue. 2. Education refresher regarding nutrition and sleep (2 x 2 hrs) based on feedback during deployment.
**Deployment periods**			
Mountain training period	3 weeks (Feb)	Mixed education/intervention with PTI:	
		1. Injury prevention and recovery applied to a technical and tactical setting.	
		2. Physical activity as a means (technical), an end (tactical), and a recovery resource (mountaineering activity during the free week-end).	
		3. Emphasis on the importance of managing physiological resource spending and acceptable pain thresholds depending on the context.	
		4. Illustration of nutrition choices depending on the type of activity.	
Operational deployment	3 months (Aug-Nov)	Interventions:	
		1. Managing nutrition in a resource-constrained environment, based on the previous lectures and workshops.	
		2. Adapting sustained operations schedule to the team set-up in terms of chronotype and sleep need;	
		3. Individual physical training schedules depending on available time and space.	
		Availability of the experts (PTI, MD, Physiotherapists, Psychologists) for reach back guidance and support.	

*Overview of the integration of the specialists' approaches described in the previous sections at the scale of one team (8 operators)*.

#### Lessons Learned

We will only summarize here the main take home messages, which readily translate to team sports as well as to the SOF environment.

##### Involvement of Experts: WYGIWYG (What You Get Is What You Give)

The main challenge for the involved experts was the availability requirements: the fact that they had to be reachable almost 24/7, extremely flexible regarding timing, and very creative to combine this support function with other job requirements (as none of the involved experts was dedicated to this target population). Indeed, the scheduled weeks for interventions changed several times, and the availability of the operators was extremely volatile (which is not surprising, considering the fact that these were the only weeks of the year where they were actually in country and without training requirements). The main positive outcome was to work with enthusiastic and highly motivated people, who adhered to the program and made it their own in a couple of months, showing rapid progress in the invested dimensions. The experts thus had to show a disposition of humility, to be willing to learn from colleagues and operators, and make themselves available. The job satisfaction, in return, was proportional to the requested investment.

##### Outcome for the Team

The main positive outcome for the team was the increased quality of the team processes, insights and self-knowledge, which made them feel stronger by having clearly identified strengths and weaknesses and the leverage points to address those. The mutual transfer of knowledge between the individual interventions, team workshops and classroom sessions to real-life situations showed a successful implementation of the selected concepts. However, they also reported a high level of frustration with feeling a “culture clash” in the unit, with obstacles to putting their new knowledge into practice (e.g., scheduling or food purchases). They thus emphasized the need, for such a program to actually be effective, to be implemented at every level of management and decision making. Considering the described psychological profile of operators in terms of overachievement, it is not surprising that, if they adhere to the programme, they would feel frustrated at being hindered from applying it in the most complete and efficient way. This issue further validates our initial systemic approach, however, functioning in an organization the size of Defense, it is impossible to decentralize some decision processes. This might be easier in a sports team environment.

##### Ethical Consideration: Care and Confidentiality

Both experts and operators emphasized the importance of one of our basic cornerstones, being the confidentiality of the process. The fact that none of the assessment results (clinical, physical or mental) were part of their official record; and the fact that they had the assurance that their results were actually their property were paramount to the success of this pilot project. In order to work on one's own functioning, it is an absolute necessity to face one's limitations, failures and weaknesses. However, in an evaluation context (e.g., regarding professional fitness qualifications, or in a sports team, regarding player trading decision making), there is no room for such openness, no room for individuals to lay open their vulnerabilities. This determines the mutual trust relationship, the absolute honesty in assessment situations (for both clinical interviews and questionnaires); and the overall feeling that the programme was an alliance between professional experts and operators to provide operators with the best possible support both on an individual and team level, which is the definition of a therapeutic alliance (e.g., Gergen, 2008). The fact that many performance management programmes in the sports world are designed or managed without this clinical background and care deontology in mind is a point of attention, as this seemed essential in the success of the endeavor. It is noteworthy that the management within both the operational unit and the military medical service never questioned this position, and never attempted to overrule it. This underscores the humanistic approach of the Belgian Defense regarding performance management in its personnel.

## Discussion

Recent developments in team training show a tendency to leave the reductionist model of “just” training every individual of the team to the maximal capacity. Rather, the team is seen as an adaptive system. The approach differs in a sense that athletes/SOF operators become part of dynamic and complex systems, thus requiring a systemic and holistic approach. In 2016, Soltanzadeh and Mooney explored systems thinking and its potential for modeling and analyzing sports team performance, underlining that, to understand the individual parts of a team, we should approach the whole and vice versa. In the current HPP program, we wanted to go one step further. To understand the perspective of every team member on this intersection between individual and team, we used a participant observation approach in order to gain information from their expertise and to preserve a co-creation between the team, the individuals and the program developers (Jorgensen, 1989; McTaggart, 1991; McIntyre, 2007; Gergen, 2008; Spradley, 2016). By applying this systemic approach, we aimed at designing a human performance program that could serve both the individual and group level of the team. As Bateson emphasized, to understand human communication and team play, we must go further than the systemic thinking of thermodynamic laws that reduce dynamic relationships to linear causality: “In this strict sense, the impact of one billiard ball upon another is subject matter for dynamics, but it would be an error of language to say that billiards balls “behave”…. we, however, are not concerned with event sequences which have this characteristic” (Bateson, 1972). We thus set out to design a human performance program aimed at overcoming the dichotomy between mental and physical performance; between care for existing injuries and performance optimization; and between individual training and team functioning.

The current paper presents its first practical application within a SOF team. First things required are a current status report and a needs assessment. A current status report is obtained based on relevant, valid and reproducible test procedures. This required key physiological and psychological parameters to be identified, in order to have tracking variables. The needs assessment required consultation, participant observation and multidisciplinary analysis, making it possibly the most labor-intensive part of the program design. The combination of that current status report; the identification of the key components to measure; and the result of the needs assessment provided us with several leads for leverage. These leads were: (i) to adopt a holistic and systemic approach; (ii) to capitalize on strength by tailoring to the level of the individual operator and support constructive coping mechanisms (recreational sports; team dynamics and partner relationships); (iii) to address the main vulnerability, being musculoskeletal injuries; (iv) to identify the necessary improvements in nutrition and sleep management.

This approach allowed us to create tailor-made training programs and provide substantiated feedback at an individual and team level. Customized training programs also bring along the advantage of being more focused, and therefore more time-efficient compared to general training programs. Within a SOF-context, time-efficiency is paramount, as operators are abroad most of the year for operational missions or technical exercises, which clearly showed in our implementation project. This is also the reason why we chose, right from the start, to develop the autonomy of operators through education and the empowerment to reach a level of self-management, with a reach back guidance of experts available. The status reports further provide the opportunity to follow-up over time. Data that are gathered on a regular basis throughout the years will allow for a more and more accurate monitoring of both the individual and the group performance level. The third and final step in our approach was the integration of the individual in the team, and to let the team evolve into a strong and competent unit, with a specific set of skills well-aligned between the members of the team. This was achieved through team training, firstly for a physical dimension, then with an educational approach, then a coaching toward autonomy in the analysis of team processes in real-life exercises and deployments. The insight brought within the team was defined by the pilot project SOF team as the most important positive outcome to the program, supporting their efficiency and allowing them insight in their own functioning, eventually empowering them to leverage more tools to support their performance.

A limitation to our holistic approach is the lack of a spiritual component. Other human performance optimization programs (e.g., Chamberlin et al., 2020) specifically address this dimension in the performance optimization approach. We deliberately left it out because of its cultural sensitivity in Belgium, where our recent history is still marked by tensions between organizations acknowledging a religious affiliations and others claiming a complete separation between state and church. Despite the fact that the Belgian Defense has a humanistic and inclusive view of pastoral care, tending to several religions and non-religious moral care, we chose to pick our battles, and start with a program where the content of the expertise would not be a matter of debate. Furthermore, whereas we believe this matter to be of utmost importance for personnel confronted with life and death decisions, it might be less relevant in the transfer to the sports science environment.

An important limitation of the current paper is that it misses the final stage of Intervention Mapping, being program evaluation. There are several reasons for that. Firstly, the program objectives were defined according to needs, not according to measurable variables. Whereas, this may be seen as a weakness from a design management point of view, we see it as a strength from an ecological validity point of view: we set out to provide support regarding actual needs of the client, not regarding program design and evaluation. The objectives we thus defined do not fit an evaluation in the usual timeline of a research and development project, for these variables have a different time resolution: injury reduction, for example, takes several years to show in prevalence numbers, especially considering the chronic feature of the diagnosed injuries in the musculoskeletal assessment. It might need a complete new generation of operators, where this performance management approach is applied right from the start, to actually show a measurable benefit in prevalence. Secondly, since one of the fundamental choices in the design and implementation of this program was to advocate first and foremost for the interests of the individual operator, it is difficult to identify in this multidisciplinary framework objective, reliable and valid variables that would represent the effect of the program as a whole. As Cameron wrote in 1963: “It would be nice if all of the data which sociologists require could be enumerated because then we could run them through IBM machines and draw charts as the economists do. However, not everything that can be counted counts, and not everything that counts can be counted.” Thirdly, our aim in the current paper is to provide a detailed description of the applied method, a “kitchen recipe” to disseminate the work from the program design as such, and the rationale behind the methodological choices.

A specificity of the current paper, which can be seen as both a strength and a limitation, is that we aimed to span the whole resolution spectrum of our program design: from conceptual and management choices to workshop floor details. We believe it shows the highest level of transparency in the process, despite being unorthodox for a scientific paper. The level of detail in the assessment and intervention description is kept to ensure the usefulness of the content to practicing professionals. The resolution adjustment this demands from the reader may hamper a smooth reading, and definitely do not make for an elegantly parsimonious structure, like we normally favor in scientific papers. Yet we are grateful for this opportunity to prove Richard Feynman wrong, who addressed this issue in his Nobel prize recipient address in 1966: “We have a habit of writing articles in scientific journals to make the work as finished as possible, to cover up all the tracks, to not worry about the blind alleys or describe how you had the wrong idea first, and so on. So there isn't any place to publish, in a dignified manner, what you actually did in order to get to do the work.”

In the approach described here, multidisciplinarity is a cornerstone of the program we designed, and definitely one of its major strengths. However, one of the lessons learned from this implementation is the “hidden cost” of multidisciplinarity in terms of workload. Multidisciplinarity is only supporting a holistic approach if every professional involved is willing to step out of his comfort zone to actively look for overlaps with the other areas of expertise. This requires communication; and time to learn: time to attend screenings and trainings from the other professionals, time for joint consultations with athletes/operators, time for self-education and research. As every manager will tell: time is money. However, the creativity, drive and achievement that was experienced by every professional involved allowed to reach the goal of designing the program in a quite tight timeframe, being 30 months from the “start from scratch” at the whiteboard to the end of the pilot project implementation in real life.

This issue of the cost of multidisciplinarity and the question of program evaluation actually tie in to a third question, which will be common to military and sports settings: how can the return on investment of such an approach be demonstrated? This issue is becoming more timely than ever: in the aftermath of the Tokyo Olympics in 2021, French president Macron famously “welcomed” the French Olympic team back with an address mentioning what he called the lack of results compared to the amount of public spending in sports (Métairie, 2021). This question of return on investment raises again the dilemma regarding what counts, and can be counted.

Return on investment brings us back to one of our basic design choices regarding the program. We set out in the framework of a care work ethic, to care for our patient/client, at the level of the individual operator/team. Regarding the translation to team sports, the same choice has to be made: does an intervention target the interests of the athlete, or the interests of the management? Considering the typical need for achievement of athletes (and operators!), these are aligned in a nominal situation, where everybody agrees on the end goal: to perform as successfully as achievable, and to bring the unachievable within reach. However, as emphasized by Malgoyre et al. (2015) in their article regarding performance enhancement in elite athletes and soldiers, at some point, the short and long-term interests of the individual and the organization may diverge regarding cost-benefit analysis of interventions. As we already discussed in the introduction: maximal performance does not equal optimal health, and many performance-enhancing choices of both elite athletes and elite soldiers may damage their long-term health and well-being. This is a conundrum to which we cannot suggest an answer, yet an issue that must remain open to discussion.

Despite the specific nature of a SOF team, as we discussed throughout the present paper, the approach described here can readily be transposed to elite team sports training. Sport scientists and practitioners will surely benefit from a further integration of dynamic constructs such as cohesion, leadership and collective efficacy, summarized as team togetherness, combined with the team's intrinsic value at the individual level (Bourbousson et al., 2019). Similarly to a SOF team, designing periodized training programs for team sports athletes poses unique challenges and difficulties. Nevertheless, Mujika et al. (2018) recently stressed that both physical and strategic periodization are useful tools for managing the heavy travel schedule, fatigue, and injuries that occur throughout a competitive season/career. Despite the different types of challenges, the result is similar for SOF teams and elite sports teams. In addition, Mujika et al. (2018), like in the current paper, put forward that psychological skills are a central component of athletic performance, and their periodization should cater to each athlete's individual needs and the needs of the team. The similar topics that have been focussed on in the current paper and in the publication of Mujika et al. (2018) stress the usefulness of comparing performance optimization programs in both fields of application (i.e., the SOF context and the team sports context) and underline that this can be crucial in order to further advance this field of research. Adopting a holistic approach in a team sports context will, like in a SOF context, allow to make more of each supporting professional's capacities, to fully utilize the team around the team. The implementation constraints in terms of cost and availability of experts are quite similar too. And contrary to the SOF setting, the team sports setting combines two features which allow for easier implementation. Firstly, there is the performance timeframe, around seasons, that could make an evaluation easier against objective external criteria. Secondly, the smaller scale of decision making levels around a sports team could make implementation faster and more efficient.

## Data Availability Statement

The original contributions presented in the study are included in the article/supplementary material, further inquiries can be directed to the corresponding author/s.

## Ethics Statement

The studies involving human participants were reviewed and approved by Institutional Review Board, UZ Brussel. The patients/participants provided their written informed consent to participate in this study.

## Author Contributions

NP, ED, MH, MS, RV, JV, JP, EN, ND, and DV participated in every stage of the program design and implementation. JV, BR, AC, GR, EL, VT, and MV provided expert knowledge back-up on the program design and regarding the manuscript redaction. NP, JV, BR, GR, EL, MV, EN, ND, and DV contributed to the manuscript. All authors reviewed and commented on the manuscript and its final version has been approved by all.

## Funding

This research was funded by grant HFM 17-05 from the Belgian Defense.

## Conflict of Interest

AC is employed by Brainwise, Ltd. The remaining authors declare that the research was conducted in the absence of any commercial or financial relationships that could be construed as a potential conflict of interest.

## Publisher's Note

All claims expressed in this article are solely those of the authors and do not necessarily represent those of their affiliated organizations, or those of the publisher, the editors and the reviewers. Any product that may be evaluated in this article, or claim that may be made by its manufacturer, is not guaranteed or endorsed by the publisher.
